# Health systems resilience in fragile and conflict-affected settings: a systematic scoping review

**DOI:** 10.1186/s13031-023-00560-7

**Published:** 2024-01-03

**Authors:** Claudia Truppa, Sally Yaacoub, Martina Valente, Giulia Celentano, Luca Ragazzoni, Dell Saulnier

**Affiliations:** 1 CRIMEDIM - Center for Research and Training in Disaster Medicine, Humanitarian Aid and Global Health, Università del Piemonte Orientale, Novara, Italy; 2 International Committee of the Red Cross, Geneva, Switzerland; 3 Center for Research in Epidemiology and StatisticS (CRESS), Université Paris Cité and Université Sorbonne Paris Nord, Inserm, INRAE, 75004 Paris, France; 4 Department for Sustainable Development and Ecological Transition, Università del Piemonte Orientale, 13100 Vercelli, Italy; 5 ETH Zürich, Institut Für Bau- Und Infrastrukturmanagement, Chair of Sustainable Construction, Zurich, Schweiz; 6 Division of Social Medicine and Global Health/Department of Clinical Sciences, Lund University, Malmö, Sweden; 7 Geneva Centre of Humanitarian Studies, Université de Genève, Geneva, Switzerland; 8 Department of Global Public Health, Karolinska Institutet, Stockholm, Sweden

**Keywords:** Health systems resilience, Health systems research, Conflict

## Abstract

**Background:**

Health systems resilience (HSR) research is a rapidly expanding field, in which key concepts are discussed and theoretical frameworks are emerging with vibrant debate. Fragile and conflict-affected settings (FCAS) are contexts exposed to compounding stressors, for which resilience is an important characteristic. However, only limited evidence has been generated in such settings. We conducted a scoping review to: (a) identify the conceptual frameworks of HSR used in the analysis of shocks and stressors in FCAS; (b) describe the representation of different actors involved in health care governance and service provision in these settings; and (c) identify health systems operations as they relate to absorption, adaptation, and transformation in FCAS.

**Methods:**

We used standard, extensive search methods. The search captured studies published between 2006 and January 2022. We included all peer reviewed and grey literature that adopted a HSR lens in the analysis of health responses to crises. Thematic analysis using both inductive and deductive approaches was conducted, adopting frameworks related to resilience characteristics identified by Kruk et al., and the resilience capacities described by Blanchet et al.

**Results:**

Thirty-seven studies met our inclusion criteria. The governance-centred, capacity-oriented framework for HSR emerged as the most frequently used lens of analysis to describe the health responses to conflict and chronic violence specifically. Most studies focused on public health systems’ resilience analysis, while the private health sector is only examined in complementarity with the former. Communities are minimally represented, despite their widely acknowledged role in supporting HSR. The documentation of operations enacting HSR in FCAS is focused on absorption and adaptation, while transformation is seldom described. Absorptive, adaptive, and transformative interventions are described across seven different domains: safety and security, society, health system governance, stocks and supplies, built environment, health care workforce, and health care services.

**Conclusions:**

Our review findings suggest that the governance-centred framework can be useful to better understand HSR in FCAS. Future HSR research should document adaptive and transformative strategies that advance HSR, particularly in relation to actions intended to promote the safety and security of health systems, the built environment for health, and the adoption of a social justice lens.

**Supplementary Information:**

The online version contains supplementary material available at 10.1186/s13031-023-00560-7.

## Introduction

Health systems resilience (HSR) gained prominence in health systems debates following the Ebola epidemic in West Africa [1, 2], with further attention paid to fostering resilience following the onset of the COVID-19 pandemic [3–5]. These debates are rapidly developing, with key concepts emerging and several theoretical frameworks proposed. Several reviews that have been performed on the topic have highlighted variety, inconsistencies, and gaps in knowledge: this can be imputed on one side to the lack of a commonly agreed definition of HSR, and on the other to the different focuses adopted in the analysis of its characteristics, which vary from a high-level health systems strengthening perspective to a pragmatic communicable disease outbreak control angle [6–13].

One critical gap emerging in the available literature is that only limited contributions to the debate come from fragile and conflict-affected settings (FCAS) [14, 15], partly due to the intrinsic challenges associated with conducting research in these contexts [16]. For the purpose of this review, we adopt the World Bank definitions of conflict–i.e., “a situation of acute insecurity driven by the use of deadly force by a group (…) with a political purpose or motivation”–and fragility–i.e., “a systemic condition or situation characterized by an extremely low level of institutional and governance capacity” [17]. The research and practitioner communities have both highlighted the necessity of expanding research on HSR in these settings [18]. Low- and middle-income countries (LMICs) are disproportionately affected by conflict and violence, which represent the main sources of both acute shocks and chronic stressors on health care systems in such contexts [19, 20]. Among the 82 countries classified as LMICs by the World Bank in 2022 [21], more than one third (29/82, 35.4%) were considered FCAS [17]. It is estimated that by 2030 this figure will further increase to two thirds of the world’s poorest communities living in contexts affected by fragility, conflict, and violence [19]. Poverty, institutional fragility, or armed conflict create a vicious cycle of stressors and dysfunctional responses in health systems, which increase the need for resilience measures.

A second critical gap is the lack of attention on the potential contributions of multiple stakeholders acting in situations of conflict or institutional fragility, either in collaboration with or independent of governmental actors [22, 23]. Most of the research conducted on HSR in these settings focuses on institutional providers such as Ministries of Health. However, the governance structure of health systems in FCAS often includes multiple, parallel–and at times poorly coordinated–health care providers, such as state and non-state actors, public and private sector providers, religious actors, and nongovernmental organisations (NGOs). For example, following the cholera epidemic in Haiti in 2011–2012, and the Ebola epidemic in West Africa in 2014–2015, critical limitations in aligning the coordination efforts of the health systems responses were observed [24–26].

Lastly, there is a lack of guidance on operationalising resilience vis-à-vis existing theoretical frameworks. Despite the debate around the definition of HSR and their characteristics, there is limited evidence on how to strengthen resilience in practice. Much of this debate has focused on the attempt to define a common framework to analyse resilience [6, 8, 10, 12, 13]. Moreover, most of the research retrospectively analyses how a specific health system reacted to a specific shock, rather than analysing its response against a measured baseline [1, 7, 8, 10, 27–29].

Resilience is increasingly referred to as “an ability, not an outcome” [30], and refers to the strategic approaches health systems can employ to achieve positive health outcomes [23, 30, 31]. These strategic approaches unfold through decision-making processes that turn theoretical concepts into operational actions. Such action unfolds through three levels of abilities or capacities: absorptive, adaptive, and transformative [32]. This lens of analysis is increasingly adopted to investigate HSR and has been recently applied to health systems research in FCAS specifically [33].

For the purpose of this review, we refer to resilience as the capacity of a health system to absorb and adapt to disruptive events in order to maintain continuity of planned, essential health services, and to reorganise and transform in response to disruptions [1, 32].

This scoping review addresses the aforementioned identified gaps, analysing the state of HSR research in FCAS. The overall aim of this review is to explore which HSR concepts have been emphasised in these contexts, in order to draw recommendations on both future research and programmatic aspects of health interventions delivered in situations of armed conflict and fragility.

This review sought to: (a) describe the conceptual frameworks of HSR used in the analysis of shocks and stresses in FCAS; (b) analyse the representation of different actors involved in health care governance and service provision to HSR in these settings; and (c) identify health systems operations as they relate to absorption, adaptation, and transformation in FCAS.

## Methods

A scoping review of scientific and grey literature published on HSR in FCAS was conducted according to the recommendations formulated in the Joanna Briggs Institute (JBI) Manual for Evidence Synthesis [34], and was reported following the Preferred Reporting Items for Systematic Reviews and Meta-Analysis (PRISMA) guidelines [35] and its extension for Scoping Reviews (PRISMA-ScR) [36] (Additional file 1: Appendix I). The study protocol was registered in the Open Science Framework (OSF) with Digital Object Identifier (DOI) 10.17605/OSF.IO/G9RWM.

### Eligibility criteria

Studies and reports had to present an explicit focus on HSR in FCAS. Any publication type was considered eligible, as the assumption that prompted a scoping review format was that there is insufficient evidence to conduct a systematic review and meta-analysis.

An explicit focus on HSR was defined as an unambiguous reference to an existing HSR framework in the objectives and/or methods of the study, or the analysis of findings in the discussion section through a HSR perspective or framework.

**Fig. 1:**
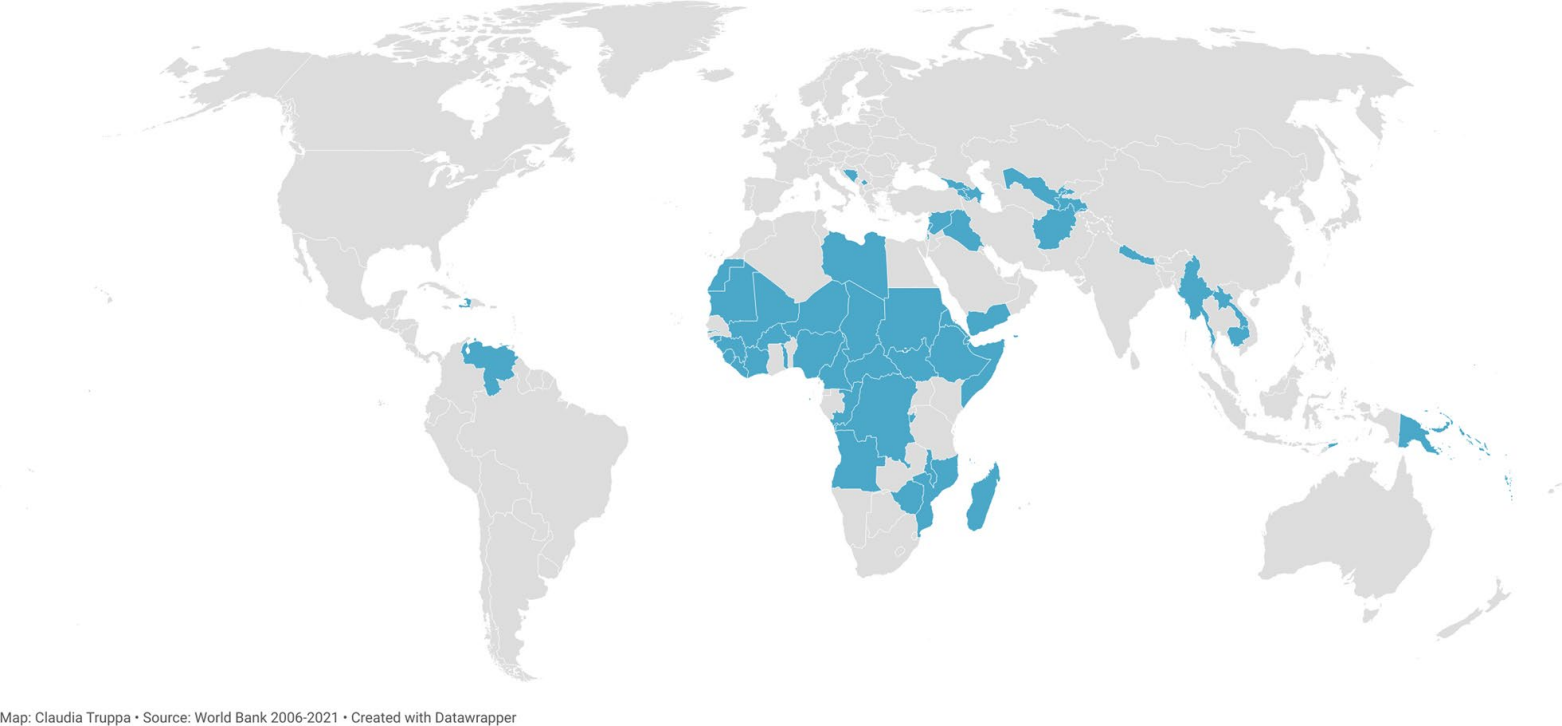
Countries and territories included in the World Bank classification of FCAS, 2006–2021

Studies and reports were included if they were conducted in FCAS per the World Bank classification [17]. A total of 61 countries have been classified as FCAS since the classification was introduced (Fig. 1 and Additional file 1: Appendix II). Studies published from 01 January 2006–the year in which the World Bank classification of FCAS was first introduced–to 31 January 2022, and written in Arabic, English, French, German, Italian, Portuguese, or Spanish, were included. Studies and reports were excluded if they explored individual psychological resilience.

### Information sources and literature search

PubMed, Scopus, and Web of Science were searched on 14 February 2022. In order to develop a full search strategy, we used the key words contained in the titles and abstracts of relevant articles retrieved during preliminary searches, as well as the index terms linked to these articles. The search strategy for each database was built with the objective of being sensitive rather than specific and included index terms and free string searches for the three key concepts under study: (1) health systems, (2) resilience, and (3) armed conflict, fragility, or violence, along with relevant synonyms (Additional file 1: Appendix III). The reference lists of all included literature and other relevant articles were also screened for additional studies. The grey literature was manually searched between March and May 2022 and included several information sources and websites of international organisations and donors, such as Evidence Aid, Research Square, Research Gate, the Health Systems in Fragile and Conflict-Affected Settings collection, as well as the websites of Médecins sans Frontières (MSF), the International Committee of the Red Cross (ICRC), the United States Agency for International Development (USAID), the World Bank, and the Foreign, Commonwealth and Development Office (FCDO), among others. All identified citations were uploaded into Zotero version 6.0.22 (Corporation for Digital Scholarship, Vienna, Virginia, USA), after which duplicates were removed.

### Study selection

Unique citations were uploaded to Rayyan for screening [37]. Following a pilot test in which the two reviewers screened 20 entries, titles and abstracts were screened independently and in duplicate for assessment against the eligibility criteria. The reviewers obtained the full texts of papers judged as potentially eligible. Seventy-three authors were contacted to request inaccessible full texts. Fifteen papers were considered not accessible as the corresponding authors did not respond to this request.

The full texts were screened for eligibility by one reviewer using a standardised and pilot-tested screening form. Calibration exercises were done monthly throughout the screening and data extraction process, with the second author independently assessing the eligibility of a random subset of articles. The disagreement rate throughout title and abstract screening was below 5%, and the final disagreement rate upon completion of screening was 4.8% (218/4538). Disagreements during full text screening were reconciled through discussion, and through the involvement of an additional reviewers whenever needed.

### Data extraction

The data extraction form was pilot tested by the two reviewers on the first five included studies to identify potential inconsistencies or gaps in the identified categories. Data was extracted using the pilot-tested data extraction form that was approved by all co-authors. The form was modified and revised as necessary during data extraction (see Additional file 1: Appendix IV). The final form included general characteristics of the papers as well as key themes related to resilience following the categorisation proposed by Macrae and Wiig [38], and as adapted to health systems by Lyng and colleagues [39]. This was considered the most appropriate categorisation for data extraction as it is explicitly aimed at exploring resilience on a theoretical and a practical level, both of which are addressed in this review. As such, it included information about the context and the phenomena of resilience, i.e., the goals and objectives, triggers, materials and resources, and mechanisms of resilience, as defined by Wiig et al. [40]. One reviewer independently assessed a subset of data extraction forms. Any disagreements that arose between the reviewers were resolved through discussion, or with the involvement of additional reviewers.

### Data synthesis

Data were charted in an Excel spreadsheet. A primarily deductive analysis was performed, following the different categories derived from the governance-centred resilience framework proposed by Blanchet et al., identifying four dimensions (knowledge, uncertainty, interdependency, and legitimacy) and three levels (absorptive, adaptive, and transformative) [32]; and the characteristics of resilient health systems described by Kruk et al. (awareness, diversity, self-regulation, integration, and adaptiveness) [1, 41]. Themes related to HSR were also analysed in relation to the World Health Organization’s (WHO) health systems framework [42]. Despite the limitations of the WHO building blocks as an analytical tool [43], they proved useful in the context of this study as they provide a widely accepted reference for thematic analysis in health systems research in general [44], and in HSR research specifically [10].

## Results

**Fig. 2:**
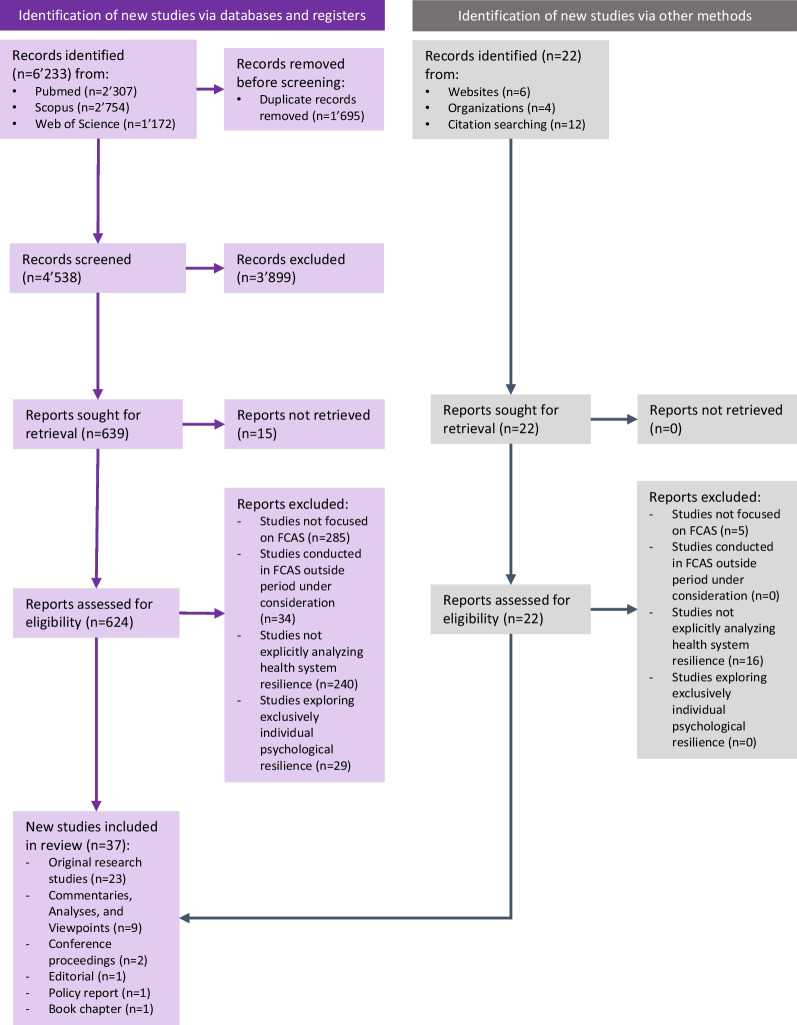
PRISMA flow diagram showing the results of the systematic scoping review on health systems resilience in fragile and conflict-affected settings

**Fig. 3:**
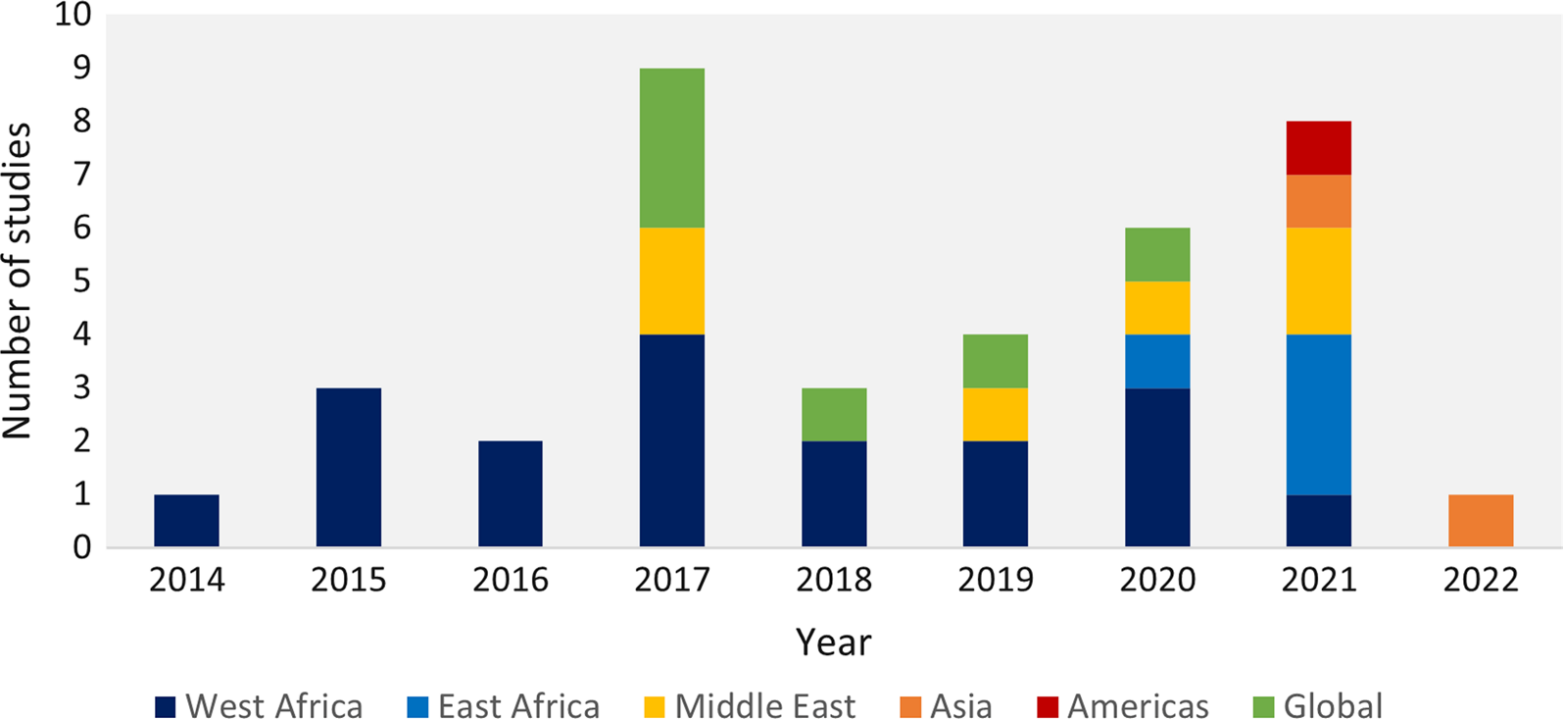
Trends of publication of the 37 studies included by year and region

Thirty-seven publications were included, of which 23 presented the results of original research studies, while the remainder were commentaries, conference proceedings, editorials, or reports (Fig. 2 and Additional file 1: Appendix V). Twenty-five publications drew from settings in sub-Saharan Africa, followed by seven studies the Middle East (Table 1 and Fig. 3).

**Table 1:** Summary of the characteristics of the 37 articles and reports included in the systematic scoping review on health system resilience in fragile and conflict-affected settings

Study characteristics	Number	References
*Year of publication*		
2014	1	[75]
2015	3	[1, 45, 53]
2016	2	[2, 46]
2017	9	[14, 41, 47, 62, 68, 70, 72, 74, 76]
2018	3	[29, 50, 54]
2019	4	[56, 58, 59, 63]
2020	6	[48, 55, 57, 60, 69, 71]
2021	8	[49, 51, 52, 64–67, 73]
2022	1	[61]
*Type of publication*		
Original research study	23	[29, 45–50, 52, 54–58, 61–68, 71, 73]
Commentary/Analysis/Viewpoint	9	[1, 2, 14, 41, 51, 53, 60, 72, 74]
Conference proceeding	2	[70, 76]
Editorial	1	[75]
Policy report	1	[69]
Book chapter	1	[59]
*Geographic setting*^*a*^		
FCAS in general	3	[54, 59, 69]
Sub Saharan Africa	25	
West Africa in general	7	[1, 2, 29, 50, 53, 75, 76]
Sierra Leone	6	[14, 58, 60, 62, 64, 68]
Liberia	6	[41, 47, 48, 60, 63, 72]
Nigeria	2	[45, 46]
Congo (DRC)	2	[65, 67]
Cameroon	1	[71]
Sudan	1	[73]
South Sudan	1	[55]
Middle East	7	
Gaza	1	[74]
Iraq	2	[66, 70]
Lebanon	3	[41, 51, 56]
Syria	1	[57]
Southeast Asia	4	
Cambodia	2	[14, 68]
Myanmar	1	[49]
Nepal	1	[61]
Latin America	1	
Haiti	1	[52]
*Type(s) of shock*^*b*^		
Infectious disease outbreak	22	[1, 2, 29, 47, 48, 50–53, 58, 60, 62–65, 68, 69, 71, 72, 75, 76]
Armed conflict and violence	17	[14, 41, 45, 46, 51, 54–57, 59, 63, 65–68, 70, 74]
Natural disaster	5	[1, 49, 52, 61, 74]
Climate hazards	2	[70, 73]
*Actors under analysis*^*c*^		
Communities	4	[48, 50, 52, 63]
Public health sector	24	[1, 2, 29, 41, 45–47, 51, 53–55, 58, 60–62, 64, 66–72, 76]
Private for-profit health sector	8	[1, 29, 49, 51, 58, 60, 67, 75]
Private non-for-profit health sector	19	[1, 14, 29, 45, 46, 49, 51, 54, 56, 57, 59, 60, 64–67, 73–75]
*Resilience framework adopted*^*d*^		
Not specified	17	[2, 51–53, 55, 59, 60, 62, 64, 68–70, 72–76]
Kruk’s characteristics of resilient health systems	7	[1, 41, 45–49]
Blanchet’s capacity-oriented resilience framework	5	[56, 57, 63, 66, 67]
Others	9	[14, 29, 49, 50, 54, 58, 61, 65, 71]

^a^Total number exceeds number of included papers as some refer to multiple countries

^b^Total number exceeds number of included papers as some refer to multiple shocks

^c^Total number exceeds number of included papers as some refer to multiple actors

^d^Total number exceeds number of included papers as some refer to multiple frameworks

### Characteristics of resilience based on Kruk et al.’s framework

Seven studies used Kruk et al.’s framework in relation to either the Boko Haram armed insurgency in Nigeria, the West Africa Ebola outbreak, or Cyclone Nargis in Myanmar [1, 41, 45–49].

At the system level, fragmentation of health actors and institutions intervening in countries affected by conflict and fragility was commonly described in relation to the framework’s characteristic of diversity. In multiple studies, parallel coordination platforms were initiated to propose a vision for the governance of the health sector [29, 50, 51]; to establish funding mechanisms [14, 29, 47]; to create a suitable health management information system [29]; and to support supply chains and service delivery [47, 50–52].

As opposed to these parallel mechanisms, integration—the capacity to bring multiple actors and systems together, aligning actions towards a common goal through effective coordination [41, 45, 53, 54]—was described as more conducive to resilience. For example, the presence of multiple actors intervening in a crisis situation is described as a source of beneficial redundancies, i.e., collateral pathways implemented in service provision to avoid interruptions of operations in case of disruptions, which supported the resilience of the health systems in northern Nigeria and in South Sudan in response to chronic violence [45, 55].

Flexibility and creativity were frequently described as important features of adaptiveness during times of crisis, particularly for the health workforce. In FCAS, flexibility and creativity were linked to increases in demand linked to population displacement [51, 56, 57] or to the scarcity of resources and means due to disrupted supply chains [58–61]. These characteristics were also described as important within the informal health workforce. For example, traditional birth attendants in Liberia created makeshift gloves from plastic bags when access to personal protective equipment was constrained at the beginning of the Ebola outbreak, in order to continue assisting home deliveries where no other services were available to the population [50].

Awareness and self-regulation were seldom discussed, and were mostly referred to in the analysis of responses to infectious disease outbreaks in West African countries [1, 41, 62]. Both concepts were introduced by Kruk et al. and concern the capacity of health systems to identify potential health hazards through adequate surveillance, and that of reacting upon alerts generated to ensure rapid response, respectively [1, 41]. In these contexts, routine data collection and rapid analysis tools have also been shown to help predict the indirect effects of an epidemic on maternal, newborn, and child mortality, which is presented as a strategy to address early the continuity of essential healthcare services [62].

### Dimension of resilience based on Blanchet et al.’s framework

In FCAS legitimacy was a central theme linked to trust (or lack thereof) from the population towards public health systems [2, 49, 51, 54, 60, 63–65]. Such mistrust towards governmental institutions, even when related to shocks that are not related to the conflict, was often described as rooted in the long experience of conflict and fragility, for example in West African countries affected by Ebola [2, 60, 63–65]. Studies that were set in countries affected by protracted crises, such as Iraq and Lebanon, also described weak trust among citizens in governments that have not ensured coverage of their basic health needs over several decades [51, 66].

The interdependence of the health system and other systems was described in three articles [2, 59, 60]. The health system was linked to systemic social variables that characterize the vulnerability of the affected population, such as poverty coupled with the lack of financial safety nets for access to healthcare [14, 29, 51, 67]. For example, findings from the ReBUILD Consortium’s research show how the protracted nature of conflict generates poverty by different pathways [14]. At the household level, the direct effects of violence on families’ revenue directly impacts the possibility of early health seeking behaviours. At the health system level, attacks on health care facilities and infrastructure increase the running costs for health provision, often without a parallel increase in the available budget [14].

Uncertainty was mainly described in relation to the unpredictability of external shocks and the internal political situation of a conflict-affected country. The safety and security of health care facilities, the healthcare workforce, and communities was a theme repeated in several studies [45, 54–57, 67, 68]. Insecurity was described in different forms: obstruction of movement due to fear of armed groups for both service users and health care workers (HCWs) in Nigeria and the Democratic Republic of Congo (DRC) [45, 67]; roadblocks and riots in Haiti [52]; collateral or deliberate damage to health infrastructure during armed conflict in Syria and Iraq [57, 66]; and psychological or physical violence against HCWs during the Ebola epidemic [60] and the COVID-19 pandemic [69].

Few studies discussed the dimension of knowledge in FCAS [29, 45, 70]. Descriptions were typically related to information flow; this was reported as disrupted due to blockages at different points, from difficulties accessing data in conflict areas [70], to the incompleteness of accessible data during crisis periods [45], the critical role of community leaders in bridging interrupted flows [45], and the need to translate available information into action [29].

### Thematic areas from other frameworks

Governance was repeatedly discussed as a central element of HSR in FCAS. Four studies conducted in response to infectious disease outbreaks integrated governance explicitly in the conceptual frameworks that they proposed [29, 58, 65, 71]. Governance was also described in studies conducted in response to conflict and violence as chronic stressors to health systems, where it was emphasised in terms of weak capacity to create a shared vision, and rigidity in the decision-making processes at the same time [14, 54].

The health care workforce was among the most extensively discussed themes in the analysis of resilience of health systems in FCAS, as it was addressed in 31 of the 37 included studies [1, 2, 14, 29, 41, 45–47, 49–54, 56–61, 63–73]. The prominence of this theme is rooted in the explicit assumption that HCWs are the “backbone of health systems” [69]. As such, their individual resilience is an important determinant of group-level resilience, which is in turn intertwined with system-level resilience: the former can deeply influence motivation, retention, and the equitable distribution of human resources in times of crises, which are seen as elements that support and enact the resilience of the health system as a whole [14, 61, 68, 69].

Service delivery was explicitly included in several frameworks used to investigate HSR in FCAS [50, 54, 58]. Most articles discussed service delivery in relation to continuity of care during crises, which was described as the overarching goal of resilient health systems in 18 of the 37 included studies [45, 46, 49, 50, 52, 53, 55–61, 65, 66, 69, 70, 73]. Quality of care, on the other hand, did not appear as an integral element of any framework, but was reported in some studies as significantly eroded and noted as a critically neglected area in the response to acute shocks and chronic stressors in FCAS [47, 50, 61, 67, 68].

The availability of medical products was widely discussed in FCAS [29, 46, 50, 51, 53, 54, 58, 59, 63, 66–68, 70, 71], with supply chains included in two HSR frameworks adopted or proposed for HSR frameworks specific to FCAS [50, 58]. Infrastructure was also an element discussed in FCAS settings. Infrastructure was included in the “4 Rs” framework adopted to explore hospital resilience in Nepal [61], and in the Health System Resilience for Emerging Infectious Diseases (HSREID) framework used by Bang et al. to describe Cameroon’s HSR during the COVID-19 pandemic [71].

### Health systems resilience concepts outside of the currently available frameworks

Four themes specifically relevant to FCAS were identified across each of the different frameworks adopted: social capital; coordination; safety and security; and structural violence.

Social capital is described by Grimm et al. in their analysis of Myanmar’s HSR during Cyclone Nargis [49]. In this context, the authors refer to the safety net represented by the informal network of civil society organisations and religious groups, who often are front-line responders, particularly in remote areas where the formal health system is virtually absent. The relationships and mutual trust across sub-systems–in particular between informal community-based networks and formal health institutions–was identified as a potential key enabler of resilience in multiple studies [14, 29, 54, 65].

Coordination and partnerships were often described as critical elements to optimize complex interactions among diverse actors intervening in the health system [50, 54, 65, 71]. The need to coordinate was described across different levels, from the integration of community representatives in institutional initiatives [1, 41, 45–50, 52], to the establishment of platforms for communication among different institutions [1, 2, 29, 47, 51, 71], and from the imperative to streamline communication channels and logistics networks [1, 29, 45, 52], to the inclusion of local and regional representatives in the coordination hubs led by global actors [41, 47, 54].

One of the most distinctive features of a resilience-informed approach for FCAS appears to be the need for effective integration of informal health providers, non-state actors, and security forces in coordination efforts, to ensure that redundancy mechanisms are established to find alternative routes for service delivery when the formal health care system cannot access the entirety of the territory or cannot respond to the entirety of health care needs [2, 29, 41, 45, 49, 50, 56]. However, Ling et al. describe the potential counterproductive effects of what is referred to as “overcoordination”. In their analysis of Liberia’s health system post-Ebola, the multiple platforms for NGO coordination ultimately immobilised the coordination process itself, rather than facilitating humanitarian action [47].

Safety and security were included as key elements of equitable primary health care (PHC) services in conflict-affected settings [54], and emerge from many of the included studies as a concern that needs to be incorporated in the planning of health services to address targeted violence against HCWs [14, 29, 52, 54, 57, 60, 66–69] and health care infrastructure [52, 54, 59, 66].

Fragility and conflict were often described as amplifiers of structural violence and societal inequities [60, 74], which unfold in a vicious cycle of vulnerability and poverty that are often not addressed in humanitarian interventions [14, 51]. For some authors, this justifies the integration of human rights, justice and equity lenses both in HSR research and operations [14, 51, 54, 74], which can be realised, for example, with the adoption of localised approaches to humanitarian aid, as was acknowledged during the Ebola outbreak in DRC [65].

### Representation of different actors’ contributions to health systems resilience in FCAS

Four types of actors were identified in the studies. We define them as follows based on the characteristics and descriptions of their goals as emerged from the review:

1. Communities, whose purpose is to ensure solidary action fostered by a sense of belonging that is rooted in a shared identity (be it geographic, ethnic, religious, or other);
2. Public health sector, whose goal is to organise and provide care for the whole population in a defined territory;
3. Private, for-profit health sector, whose objective is financial gain through the provision of health care services; and
4. Private non-for-profit health sector, including NGOs, United Nations (UN) agencies, the Red Cross Red Crescent (RCRC) Movement, and charities, whose action is oriented towards the facilitation of access to care without financial gain.

### Communities

Communities were explicitly analysed in four studies [48, 50, 52, 63], but their role and contribution to HSR was discussed or advocated for in almost all of the included papers. They were seen as key agents in enacting HSR, and not only as passive recipients of health services [1, 48].

The most commonly described role of communities in relation to resilience was through cohesion and social capital [1, 2, 45, 49, 63, 66, 71, 72], which were also postulated as sources of resilience for the health system itself [14, 41, 48, 59]. The interdependency between community resilience and HSR was described as emerging from trust (or lack thereof) between the two [41, 50, 53, 60, 63, 65, 68, 69, 75]. Community health workers (CHWs), an integral element of the community but also of the health workforce, appeared to be the interface between the health system and the society it serves. Strategies aiming at strengthening their role were often described as enablers of trust between communities and health institutions [50, 52, 53].

Community engagement was a key approach to overcome the multiple barriers to accessing health care services that conflict can create. Particularly in the studies conducted on the effect of the Ebola outbreak on the fragile West African health systems, community engagement was considered a pivotal element that ultimately helped to contain the epidemic [41, 45, 46, 50, 76]. It was also described in other settings, such as Myanmar, as an opportunity to create alternative sources of emergency relief provision when the formal health system does not have the capacity–or willingness–to intervene [49]. Community engagement was also postulated as a powerful tool to strengthen the accountability of health institutions [1, 41, 45, 46, 48, 54, 56, 64, 65], in that it could bridge the misalignment that can arise between community and government priorities [47, 54].

### Public health sector

Twenty-four of the studies focused on the public sector, which was acknowledged as the most prominent service provider in times of crises, and often the last resort for the most vulnerable populations [51, 54, 58].

The most critical aspects discussed in relation to the public sector were transparency and legitimacy. Multiple studies referred to the challenges faced by local public health institutions in ensuring coverage of essential health care services prior to conflict, often due to a scarcity of resources [2, 46, 51, 53]. When a humanitarian crisis occurred difficulties were described in absorbing or transparently allocating the increased influx of funding that was made available [29, 50, 54, 71].

Inefficient management of a crisis response was seen as fostering mistrust towards local governments in general and towards health authorities in particular [2, 47, 49, 60, 64]. In some cases, the source of mistrust was in the perceived or documented corruption of local institutions [29, 50, 54, 65]. Good governance, based on knowledge of local communities and regular communication with their leadership, appeared to be associated with better coordination and an increased perceived legitimacy of institutions, as it promotes responses that are relevant and aligned with the country’s priorities [1, 45, 46, 63].

### Private, for-profit health sector

The private, for-profit sector was included in eight studies [1, 29, 49, 51, 58, 60, 67, 75] but was never the exclusive focus of analysis. The sector was discussed in relation to its complementarity or antagonism with other sectors. This sector’s contribution to HSR in FCAS appears to lack consensus and was described in conflicting terms. Some authors acknowledge its key role as an important source of redundancy to ensure continuity of service provision in response to shocks [29, 41, 49, 70], while others underline how it could instead be the first actor interrupting continuity of health care provision in times of crises [58], or attempting to influence a country’s health policies and priorities, undermining efforts to achieve universal health coverage (UHC) [51].

### Private, non-for-profit health sector

This sector was often analysed in parallel to that of the public systems they often aim to support [1, 14, 29, 45, 46, 49, 51, 54, 56, 57, 59, 60, 64–67, 73–75], except for two studies that specifically assessed the case of the UN Relief and Works Agency for Palestinian Refugees in the Near East (UNRWA) responses to the Syrian conflict and displacement in the Middle East [56, 57].

International humanitarian actors were frequently described in positive terms as key partners of local health systems in supporting and maintaining continuity of health care service provision [51, 56, 57, 66] or in increasing their quality [46, 58, 59, 73]. However, two papers discussed the need to consider power imbalances between international organisations and local institutions, which can often lead to misaligned priority setting exercises during crises, often led by the former without a substantial empowerment of the latter [14, 76]. The misalignment was attributed by the authors to the short-term funding cycles that characterise financing during humanitarian emergencies. Some authors argue for longer-term approaches, along the so-called “humanitarian-development-peace nexus”, to increase the predictability of funding and hence coherence of programming and ultimately systems strengthening [14, 51, 59, 73]. They argue that such approaches can result in a meaningful partnership, in which priorities are co-identified and responses co-designed rather than being unilaterally imposed [59, 73].

### Absorptive, adaptive, and transformative health systems operations in FCAS

Among the included studies, absorptive operations were most frequently described for shocks and stressors in FCAS, while adaptive and transformative actions were less discussed.

Health operations were described across seven domains: safety and security; society; systems; stocks, supplies and other inputs; space and built environment; staff; and services (Additional file 1: Appendix VI).

#### Absorptive operations

Absorptive operations were intended as pre-existing policies and processes that support a health system in the immediate response to a crisis [32]. Decentralised decision-making was postulated as a powerful governance model that sustained HSR in FCAS. There were few examples of the successful delegation of power, limited to health systems with clear boundaries such as the UNRWA-supported health systems in Syria, Lebanon, and Jordan [56, 57]. FCAS were commonly described as characterized by a “command and control” leadership model, that was even more centralised in situations of crisis. Decentralisation was discussed as a missed opportunity for resilience in FCAS [2, 29, 46, 49, 66, 71].

The coordination efforts of health institutions with security forces appeared to be critical to enhance the safety of health care infrastructure and staff [55, 57, 66]. In countries affected by protracted crises, such as Syria and Iraq, the existence of pre-existing communication mechanisms between the two enhanced the health system’s capacity to maintain safety and security for HCWs and to protect access to care for affected populations [57, 66]. The same effect on enhancing resilience was hypothesised in South Sudan, where better outcomes in terms of health service continuity were described in central states, where both security forces and health institutions are more present and have already established communication channels [55].

Staff commitment and motivation were frequently described as drivers of resilience. They were linked to solidarity with local communities, fostered by a profound sense of belonging to, and social cohesion with, the same community affected by conflict and violence [1, 2, 45, 52, 56–58, 60, 61, 63, 69, 70]. Psychosocial support initiatives directed at HCWs were commonly proposed activities aimed at supporting their individual and group resilience, and hence broader HSR [52, 57, 60, 66, 67, 69]. Task-shifting was proposed as another absorptive operation to strengthen the capacity of CHWs, social workers, and nurses and midwives to take higher level responsibilities during crises. This was thought to establish potential redundancies for continuity of service provision when internal or external “brain drain” limits the availability of a more highly skilled workforce [54, 56, 57, 57, 66, 67, 69].

#### Adaptive operations

Adaptive operations were considered adjustments in the organisational structure that allowed for a reallocation of resources to respond to the increase in demand for health care services [32].

For the built environment in which health care service provision takes place, the establishment of temporary structures for surge capacity was frequently described for FCAS. The approach was criticised by the authors describing it [47, 63], while others advocated strengthening existing infrastructure [57, 61].

Both financing and logistics were noted as central to adaptivity during times of crises [50, 51, 59]. For example, removing financial barriers at the population level included adopting subsidised packages of care, such as those implemented by the PHC Network in Lebanon [51]. At the system level, pooling humanitarian funds was described as an intervention that helped facilitate coordination and strengthen accountability [29, 41, 55, 70].

The logistics of medical product procurement and supply was often discussed for humanitarian organisations, whose role was proposed in terms of support to FCAS supply chains and expansion of their contingency stocks [59, 66, 71]. The UNRWA experience in Jordan and Lebanon suggested an additional option, which consisted of diversifying suppliers to establish redundancy in procuring drugs and consumables [56].

Mobile clinics were a common adaptive programmatic approach for ensuring continuity of service delivery [49, 57, 67]. Improving clinical protocols and procedures in general, and infection prevention and control measures in particular, were also often referred to, particularly during infectious disease outbreaks [47, 71]. Vertical approaches to service delivery appeared to be common practice, with only one example of adaptation by integrating essential health care services within a vertical programme from MSF during the Ebola outbreak in DRC [65].

#### Transformative operations

Transformative operations can be understood as activities and processes adopted by health actors that require a restructuring of their internal and external processes based on the emerging properties of the systems in which they operate [32]. In FCAS, these are the least documented types of operations.

Health information management system (HIMS) was described as the domain that mostly benefits from transformation [2, 46, 54, 56, 62]. Transformative innovations were sometimes introduced during a crisis, creating challenges in terms of workload for health staff, as was the case for UNRWA in Lebanon [56]. The experience of the same organisation in Jordan has suggested, for example, that investing in digital HIMS prior to a shock can increase absorptive capacity when a crisis erupts [56]. A proposed transformation in information systems that was proposed for FCAS was the strengthening of data management not only for surveillance purposes, but also to support the construction of an evidence base for interventions specifically supporting HSR through operational research [14, 50, 54].

The built environment in which health care delivery takes place is often exposed to different types of hazards in FCAS, from pervasive violence to extreme weather events. To address the increased health needs determined by a sudden shock to health systems, the set-up of temporary infrastructure (mainly in the form of tents), where infrastructure is intended in its material form [77], is a commonly described approach. However, some authors suggest that resiliency-based *ex-novo* construction of permanent infrastructure, or rehabilitation of non-functional health facilities should be preferred as transformative approaches contributing to system level resilience [66, 73].

At the societal level, several authors argued for the integration of a social justice lens into HSR strengthening efforts to lead to transformation [51, 54, 74]. Investing in community cohesion during peaceful periods to build trust between institutions and their populations was discussed, as well as to foster community resilience as the backbone of any frontline response when a crisis occurs [47–50, 53, 54, 56, 60, 63, 71, 75]. Community engagement was documented as a way to foster a sense of ownership of FCAS institutions’ responses to shocks [41, 47, 48, 50, 76]. While this would most likely increase the absorptive or adaptive capacity of countries affected by crises, in the majority of cases it is a more radical transformation in the societal matrix that is described as needed.

## Discussion

This scoping review suggests that it might be useful to analyse HSR in FCAS from a capacity-oriented perspective and through a governance-centred framework [32], as this is being increasingly used to unpack the intricacies of complex adaptive systems such as health systems in humanitarian crises [10, 44]. Such an approach could be particularly helpful to investigate the absorptive, adaptive, and transformative actions of humanitarian organisations and the private sector. These two groups of actors are less represented in the literature as compared to the public health sector, and it is of paramount importance to understand how they can contribute to the strengthening of HSR in these settings.

The operations that support HSR appear to unfold across seven domains of interventions: system governance, safety and security, society, staff, inputs of supplies and funds, service delivery, and physical infrastructure. Our review shows that the domains most commonly described in the analysis of health systems responses to different types of shocks and stressors are those related to health systems governance, human resources, supplies and financing, and service delivery, regardless of the type of trigger being studied. In situations of armed conflict and violence, safety and security, infrastructure, and social capital emerge as elements of critical importance, and yet are under-investigated.

Despite growing interest in the topic, HSR remains largely underexplored in FCAS. The majority of HSR research in these contexts has been conducted in response to infectious disease outbreaks on government health systems, rather than explicitly investigating the compounding effects of multiple stressors (economic, societal, political) that determine fragility and instability. A recent review on the responses to acute shocks in FCAS conducted by Thu et al. found similar results, with 42 of the 60 studies focused on infectious disease triggers [33].While some of the findings from FCAS echo what is described in LMICs in general, other results emerged as new and specific to these contexts [78].

### From social capital to decentralization, and from coordination to information flow: themes shared between FCAS and LMICs

The global “crisis of trust” that has followed the COVID-19 pandemic is representative of a long standing issue affecting FCAS [79], including: community mistrust towards government health institutions and their capacity to respond to the community’s needs [41, 51, 54, 60, 65, 66]; international organisations’ mistrust towards local institutions triggered by a lack of transparency while managing crises [49]; and government and citizens scepticism towards international organisations vis-à-vis allocating funds, prioritising actions, and managing resources [50, 65]. Trust building is a key action to enable resilience, and CHWs, as an interface between communities and health institutions, could be key to enhancing trust-building efforts. Their potential role has been extensively described by Sripad et al. in Haiti [52] and by Miller et al. in Ebola-affected West African countries [50], echoing what has been described in the literature from LMICs more broadly [80]. However, scholars still debate how to sustainably engage CHWs in resilience building efforts, as different models of support have been described to enhance their commitment and motivation [81, 82]. More research is needed to document successful and sustainable ways of engaging with CHWs, and of strengthening the privileged relationship they often have with affected populations [83, 84].

In FCAS, decentralisation is commonly referred to as a missed opportunity to bolster resilience [2, 29, 33, 46, 49, 66, 71]. Decentralised health system governance has been described as a successful although complex strategy to improve health outcomes in LMICs [85–87]. In FCAS, studies still debate whether centralisation is a cause or an effect of fragility, notwithstanding evidence that decentralisation can defuse conflict [88–90]. In the health care sector, centralised support is a common modality of UN-led humanitarian health interventions [91]. There are limited examples of successfully decentralised humanitarian assistance in the health sector during crises, such as the UNRWA response to the Syrian crisis [56, 57] or the MSF response to the “migrant crisis” in Europe [92]. In both cases, decentralisation is described as internal, within the power structures of the organisations, rather than externally oriented to empower decentralised local institutions. Development organisations often target state-building, arguing that strong central governments can consolidate national sovereignty in post-conflict settings [93]. This is a more contested approach among humanitarian actors [94]. Localisation of humanitarian aid, however, could support decentralised governance, representing a powerful strategy to bridge the humanitarian-development-peace nexus [95, 96]. Some humanitarian organisations have a specific mandate to rapidly deliver life-saving humanitarian assistance, and to do so they often need to be granted access to victims of armed conflict by highly centralised and hierarchical governments. Whether they can play a role in negotiating decentralised governance models for HSR while maintaining neutrality, impartiality and independence, is an ongoing debate [94].

Another theme of HSR that FCAS share with LMICs more broadly is the need for strong coordination mechanisms among the diverse institutions that play a role in the health system. In the studies included in this review, coordination is described as clearly identifying the actors involved in health operations, defining their roles, responsibilities, and accountabilities, and establishing space to communicate and share information among them [1, 41, 51, 53, 54, 72]. Such spaces in FCAS often appear to be “fragmented” [14, 51] or “chaotic” [47] due to the irreconcilable agendas of the different state, non-state, and international actors involved. In addition to involving multiple actors, highly centralised health systems are described as an impediment to effective information sharing, coordination, and accountability between the different stakeholders involved in a crisis response, such as during Cyclone Nargis in Myanmar [49] or the response to the COVID-19 pandemic in Cameroon [71].

While information flow is key to coordination and HSR, health information systems have not been well-researched in FCAS, as also noted globally [10]. In this sense, knowledge and awareness appear to be overlooked dimensions for HSR. The fundamental role of information sharing and establishing feedback loops within and across systems has been recognised as a key determinant of HSR during the COVID-19 pandemic [78]. In FCAS, identifying potential sources of information needs to be a diverse and inclusive process because of the multiplicity of actors involved, including communities and non-state actors [2, 29, 54, 65]. The challenge that remains is how to improve information flow across different levels of the formal and informal health system, and between these systems and the communities they serve, to ensure that all relevant actors are included in improved decision-making processes [41, 51, 63].

### Safety, security, and structural violence: Specific features for health systems in FCAS

This review provides insight on two additional aspects that could strengthen existing HSR frameworks and tailor them to operational needs in FCAS [78]: (1) the safety and security of communities, HCWs, health infrastructure, and medical goods, and (2) structural violence.

Deliberate targeting of health care in conflict settings is an increasingly documented phenomenon [97–99], for which the available evidence most likely under-estimates the full extent of the problem [100]. The ICRC defines violence against health care as “violence, in all its forms, that impedes, prevents or otherwise impacts the effective delivery and/or receipt of healthcare” [101]. As such it can affect patients, health care providers, infrastructure, means of transportation for both patients and medical products, and health information management systems. For health care providers in particular, the chronic threat to their safety can have a deep impact on their physical and mental health. The individual psychological resilience of HCWs, as the backbone of any health system, can ultimately impact the resilience of the system as a whole, as is increasingly described in FCAS [52, 57, 60, 69].

The structural violence underpinning social and political dynamics in conflict-affected settings generates inequities and power asymmetries [54, 74]. Using a social justice lens to capture the close relationship between health equity and gender equity has been widely discussed globally [102, 103] and recently advocated for in FCAS [104, 105]. Adopting this lens would help capture power imbalances in decision-making that hinder resilience, which is increasingly recognised as an overlooked element in health system and policy research in general [30], and in FCAS in particular [33, 78, 106]. It would integrate elements that are broadly referred to in HSR literature in FCAS, providing a cohesive, overall umbrella across frameworks that could include the legitimacy of health institutions [49, 52, 65], community trust and empowerment [29, 48, 54], values and beliefs that underpin choices at community level [2, 29], the moral context in which HCWs operate when they are confronted with often unbearable choices in a context with scarce resources [60], social capital [45, 49], and the need to strike the right balance across the range of power and interests at stake in FCAS [1, 41, 45].

Power dynamics and their influence on decision-making emerge as critical processes influencing HSR. As such they can support or hinder resilience across a continuum from individuals and communities, and from HCWs to public and private health institutions. Each actor’s agency, and hence capacity for resilience, is argued to be dependent upon and of influence on the next actor, across “vertical interdependences” within the health system [78]. While in HSR research in general there is often a lack of focus on how the resilience of one actor can support the resilience of the broader system [48, 107–112], in FCAS this appears to be a key area of focus [14, 50, 58, 60, 61, 63, 68, 69]. HCWs, often being both members of the community and representatives of the health system, emerge overwhelmingly across the papers included in this review as a critical element bridging resilience from the community to the health system level. This appears to be facilitated in particular by their commitment and motivation, rooted in both their professional ethics and in their sense of belonging to the communities they serve [29, 45, 49, 52, 53, 56–58, 61, 64, 66, 69, 70, 72].

### The role of the private sector for HSR: an open debate

The private sector was described as both a barrier and a facilitator to resilience in FCAS. Across geographical settings, from Liberia, Sierra Leone and Guinea, to Iraq and Myanmar, and in response to a variety of both acute shocks and protracted crises, private for-profit health care providers are depicted as a useful source of redundancy for continuity of service provision [29, 41, 49, 70]. On the other hand, in Lebanon they are considered a hindrance to achieving the UHC goals of the public sector as they promote a heavily hospital-centred model and divert resources from the public PHC sector. This negatively affects access to care for the most vulnerable people affected by a vicious cycle of increasing costs and decreasing sustainability [51]. Similarly, the private, non-for-profit sector can also be seen as a resource for strengthening the resilience capacities of FCAS health systems [51, 56, 57, 66] or as an obstacle to it [65, 74]. No clear pattern emerges, and more in-depth research is needed to specifically describe the pathways by which humanitarian and development organisations can support a country’s effort to strengthen the resilience of their health systems. Systems thinking methods, such as those employed in the UNRWA response to the Syrian conflict and displacement [57], or the northern Nigeria health system’s response to the Boko Haram insurgency [45], are promising tools to understand the complex interactions between multiple actors and interventions, and could be more broadly adopted to describe the role of the private for-profit and humanitarian sector in health systems in FCAS [23, 113].

### Health systems in FCAS can absorb and adapt, but how can they learn to transform?

Regardless of the specific type of sector analysed, the types of health operations described in FCAS are mainly absorptive and adaptive, in line with what has been described both globally [78] and in FCAS [33]. Some authors have suggested that the weak focus on transformation is built in to the way resilience is conceptualised, as the learning and changing aspects of resilience are often overlooked both in the definition and documentation of practices [78]. It could also be argued that the time span of observation of events in HSR research is too short to detect meaningful changes in the structures of health institutions and systems.

It is worth highlighting how operations do not appear to be intrinsically either absorptive or adaptive, but rather in terms of their relation to the natural history of a health system response to shocks and stressors. For example, task-shifting is commonly described as an absorptive action when already in place, and an adaptive initiative when it is designed after the onset of a crisis to overcome staff shortages due to either internal or external “brain drain” in situations of conflict or violence, creating redundancies in service provision that can support their continuity [54, 56, 66–69]. Another example of action that can be both absorptive and adaptive is the presence of strong, diverse and inclusive HIMS, and in particular systems able to integrate informal and non-traditional sources of information. It is suggested that pre-existing seamless information flow and feedback mechanisms positively sustain evidence-informed decision-making, while where this is implemented in the immediate response to a sudden shock it facilitates rapid adaptations [51, 54, 57].

There are very limited documented operations that led to transformation within the health system in which they were conducted, and those reported are more commonly taking place within relatively controlled systems, either because of their clearly defined mandate such as in the case of UNRWA [56, 57], or because of the closed setting being described, as in the case of a hospital built by the Italian NGO Emergency in Sudan [73]. Changes in health programme design and the introduction of new health programmes to respond to emerging needs are the most commonly described transformative operations taking place in FCAS. For example, UNRWA introduced mental health services for conflict-affected Palestinian refugees in Syria [57] and a non-communicable diseases (NCD) service package for Palestinian refugees from Syria fleeing to Lebanon and Jordan [56]. Future HSR research in FCAS will need to document how continuity of service provision for this type of services can be ensured, knowing the existing challenges related to harmonising approaches across countries and humanitarian organisations [114].

### Strengths and limitations

A major strength of this review was the broad search strategy that captured publications in seven languages, although all the included studies were published in English. A limitation was the narrow inclusion criteria, which excluded some health system research specific to FCAS that did not explicitly report on HSR in the paper. However, we do not perceive that these narrow criteria compromised the validity of our findings, as assessing implicit resilience was beyond the scope of this review. Another limitation consisted of the impossibility to granularly differentiate between high-intensity conflict, medium-intensity conflict, and high levels of institutional and social fragility. Given that these nuances have been formally introduced since 2020, it would have not been possible to retrospectively assign these categories with rigor and certainty to countries included before that date in the harmonized list [17]. Lastly, we did not adopt a ‘living’ approach for the review due to limited resources, which may have been a more appropriate choice due to the increasing volume of HSR literature [115]. Living systematic reviews are currently recommended primarily for experimental studies where up to date treatment recommendations need to be formulated [116, 117]. Therefore, this is not expected to impact the utility and validity of our findings, as the aim of this review is to contribute to a growing debate, rather than to formulate operational recommendations.

## Conclusions

Adopting a HSR lens for health systems research in FCAS emerges as an opportunity to bridge the gap between theory and practice in the analysis, planning, delivery, and evaluation of health interventions in settings affected by conflict and violence. As already suggested by Kruk et al., HSR can bring coherence across multiple perspectives, unify several health agendas under a unique umbrella, and ultimately support bridging the gap between conceptual frameworks and their operationalisation [41].

We recommend that future implementation research on HSR be guided by the governance-centred framework for HSR, as among the studies included in this review it had the utility of explaining legitimacy and interdependence in FCAS health systems, because it accounted for the coordination that is common when multiple actors intervene in response to a protracted crisis. Future research should particularly focus on the specific role of private actors–both for-profit and non-for-profit–intervening in support of public health systems in humanitarian settings, with particular emphasis on the strategic absorptive, adaptive, and transformative operations implemented to strengthen HSR in these contexts. These operations unfold across seven domains, of which three emerge as specific aspects of HSR in FCAS; these are safety and security, the built environment for health, and the adoption of a social justice lens.

Considering FCAS as host to complex adaptive systems, we recommend the adoption of systems thinking research approaches in these contexts. Such approaches will contribute to the identification of leverage points to promote the necessary social and institutional transformations needed to strengthen HSR where it is threatened the most.

### Supplementary Information

**Additional file 1. Appendix I:** PRISMA ScR checklist. **Appendix II:** Countries and territories included in the World Bank classification of Fragile and Conflict-Affected Situations from 2006 to 2021. **Appendix III:** Search strategy. III.a Search strategy adopted in PubMed. III.b Search strategy adopted in Scopus. III.c Search strategy adopted in Web of Science. **Appendix IV:** Data extraction tool. **Appendix V:** Summary of the characteristics of the 37 articles and reports included. **Appendix VI:** Absorptive, adaptive and transformative operations for HSR in FCAS.

## Data Availability

All data that support the study findings are provided in the Supplementary Material.

## Abbreviations

CHWs: Community health workers
COVID-19: Coronavirus disease 2019
DOI: Digital object identifier
DRC: Democratic Republic of Congo
FCAS: Fragile and conflict-affected settings
HCWs: Health care workers
HIMS: Health information management system
HSR: Health systems resilience
HSREID: Health system resilience for emerging infectious diseases
iCCM: Integrated community case management
ICRC: International committee of the red cross
JBI: Joanna briggs institute
LMICs: Low- and middle-income countries
MSF: Médecins sans frontières
NCDs: Non-communicable diseases
NGOs: Non-governmental organisations
OSF: Open science framework
PHC: Primary health care
PRISMA: Preferred reporting items for systematic reviews and meta-analysis
RCRC: Red cross red crescent
UHC: Universal Health Coverage
UN: United Nations
UNRWA: UN Relief and Works Agency for Palestine refugees in the Near East
WHO: World Health Organisation
