# Supplementary material for: Health systems resilience in fragile and conflict-affected settings: a systematic scoping review
Source: Confl Health. 2024 Jan 3;18:2. doi: 10.1186/s13031-023-00560-7 (PMC10763433; doi:10.1186/s13031-023-00560-7)
Supplement: Supplementary file 1 — Additional file 1. Appendix I: PRISMA ScR checklist. Appendix II: Countries and territories included in the World Bank classification of Fragile and Conflict-Affected Situations from 2006 to 2021. Appendix III: Search strategy. III.a Search strategy adopted in PubMed. III.b Search strategy adopted in Scopus. III.c Search strategy adopted in Web of Science. Appendix IV: Data extraction tool. Appendix V: Summary of the characteristics of the 37 articles and reports included. Appendix VI: Absorptive, adaptive and transformative operations for HSR in FCAS. [file 13031_2023_560_MOESM1_ESM.docx]

# Supplementary Material

## Appendix I. PRISMA ScR checklist

| **SECTION** | **ITEM** | **PRISMA-ScR CHECKLIST ITEM** | **REPORTED ON PAGE #** |
| --- | --- | --- | --- |
| **TITLE** | | | |
| Title | 1 | Identify the report as a scoping review. | 1 |
| **ABSTRACT** | | | |
| Structured summary | 2 | Provide a structured summary that includes (as applicable): background, objectives, eligibility criteria, sources of evidence, charting methods, results, and conclusions that relate to the review questions and objectives. | 3-4 |
| **INTRODUCTION** | | | |
| Rationale | 3 | Describe the rationale for the review in the context of what is already known. Explain why the review questions/objectives lend themselves to a scoping review approach. | 5-6 |
| Objectives | 4 | Provide an explicit statement of the questions and objectives being addressed with reference to their key elements (e.g., population or participants, concepts, and context) or other relevant key elements used to conceptualize the review questions and/or objectives. | 6-7 |
| **METHODS** | | | |
| Protocol and registration | 5 | Indicate whether a review protocol exists; state if and where it can be accessed (e.g., a Web address); and if available, provide registration information, including the registration number. | 7 |
| Eligibility criteria | 6 | Specify characteristics of the sources of evidence used as eligibility criteria (e.g., years considered, language, and publication status), and provide a rationale. | 7 |
| Information sources | 7 | Describe all information sources in the search (e.g., databases with dates of coverage and contact with authors to identify additional sources), as well as the date the most recent search was executed. | 8 |
| Search | 8 | Present the full electronic search strategy for at least 1 database, including any limits used, such that it could be repeated. | Appendix III |
| Selection of sources of evidence† | 9 | State the process for selecting sources of evidence (i.e., screening and eligibility) included in the scoping review. | 8-9 |
| Data charting process‡ | 10 | Describe the methods of charting data from the included sources of evidence (e.g., calibrated forms or forms that have been tested by the team before their use, and whether data charting was done independently or in duplicate) and any processes for obtaining and confirming data from investigators. | 9-10 |
| Data items | 11 | List and define all variables for which data were sought and any assumptions and simplifications made. | Appendix IV |
| Critical appraisal of individual sources of evidence§ | 12 | If done, provide a rationale for conducting a critical appraisal of included sources of evidence; describe the methods used and how this information was used in any data synthesis (if appropriate). | Not applicable |
| Synthesis of results | 13 | Describe the methods of handling and summarizing the data that were charted. | 9-10 |
| **RESULTS** | | | |
| Selection of sources of evidence | 14 | Give numbers of sources of evidence screened, assessed for eligibility, and included in the review, with reasons for exclusions at each stage, ideally using a flow diagram. | 8-9 |
| Characteristics of sources of evidence | 15 | For each source of evidence, present characteristics for which data were charted and provide the citations. | Figure 2 |
| Critical appraisal within sources of evidence | 16 | If done, present data on critical appraisal of included sources of evidence (see item 12). | Not applicable |
| Results of individual sources of evidence | 17 | For each included source of evidence, present the relevant data that were charted that relate to the review questions and objectives. | Appendix V |
| Synthesis of results | 18 | Summarize and/or present the charting results as they relate to the review questions and objectives. | 10-22 |
| **DISCUSSION** | | | |
| Summary of evidence | 19 | Summarize the main results (including an overview of concepts, themes, and types of evidence available), link to the review questions and objectives, and consider the relevance to key groups. | 22-28 |
| Limitations | 20 | Discuss the limitations of the scoping review process. | 28 |
| Conclusions | 21 | Provide a general interpretation of the results with respect to the review questions and objectives, as well as potential implications and/or next steps. | 28-29 |
| **FUNDING** | | | |
| Funding | 22 | Describe sources of funding for the included sources of evidence, as well as sources of funding for the scoping review. Describe the role of the funders of the scoping review. | 31 |

## Appendix II: Countries and territories included in the World Bank classification of Fragile and Conflict-Affected Situations from 2006 to 2021

| **Country / Territory** | **Year** | | | | | | | | | | | | | | | | |
| --- | --- | --- | --- | --- | --- | --- | --- | --- | --- | --- | --- | --- | --- | --- | --- | --- | --- |
|  | **2006** | **2007** | **2008** | **2009** | **2010** | **2011** | **2012** | **2013** | **2014** | **2015** | **2016** | **2017** | **2018** | **2019** | **2020** | **2021** | **2022** |
| Afghanistan |  |  |  |  |  |  |  |  |  |  |  |  |  |  |  |  |  |
| Angola |  |  |  |  |  |  |  |  |  |  |  |  |  |  |  |  |  |
| Armenia |  |  |  |  |  |  |  |  |  |  |  |  |  |  |  |  |  |
| Azerbaijan |  |  |  |  |  |  |  |  |  |  |  |  |  |  |  |  |  |
| Bosnia & Herzegovina |  |  |  |  |  |  |  |  |  |  |  |  |  |  |  |  |  |
| Burkina Faso |  |  |  |  |  |  |  |  |  |  |  |  |  |  |  |  |  |
| Burundi |  |  |  |  |  |  |  |  |  |  |  |  |  |  |  |  |  |
| Cambodia |  |  |  |  |  |  |  |  |  |  |  |  |  |  |  |  |  |
| Cameroon |  |  |  |  |  |  |  |  |  |  |  |  |  |  |  |  |  |
| Central African Republic |  |  |  |  |  |  |  |  |  |  |  |  |  |  |  |  |  |
| Chad |  |  |  |  |  |  |  |  |  |  |  |  |  |  |  |  |  |
| Comoros |  |  |  |  |  |  |  |  |  |  |  |  |  |  |  |  |  |
| Congo Brazaville |  |  |  |  |  |  |  |  |  |  |  |  |  |  |  |  |  |
| Cote d'Ivoire |  |  |  |  |  |  |  |  |  |  |  |  |  |  |  |  |  |
| Democratic Republic of Congo |  |  |  |  |  |  |  |  |  |  |  |  |  |  |  |  |  |
| Djibouti |  |  |  |  |  |  |  |  |  |  |  |  |  |  |  |  |  |
| Eritrea |  |  |  |  |  |  |  |  |  |  |  |  |  |  |  |  |  |
| Ethiopia |  |  |  |  |  |  |  |  |  |  |  |  |  |  |  |  |  |
| Gambia |  |  |  |  |  |  |  |  |  |  |  |  |  |  |  |  |  |
| Georgia |  |  |  |  |  |  |  |  |  |  |  |  |  |  |  |  |  |
| Guinea |  |  |  |  |  |  |  |  |  |  |  |  |  |  |  |  |  |
| Guinea-Bissau |  |  |  |  |  |  |  |  |  |  |  |  |  |  |  |  |  |
| Haiti |  |  |  |  |  |  |  |  |  |  |  |  |  |  |  |  |  |
| Iraq |  |  |  |  |  |  |  |  |  |  |  |  |  |  |  |  |  |
| Kiribati |  |  |  |  |  |  |  |  |  |  |  |  |  |  |  |  |  |
| Kosovo |  |  |  |  |  |  |  |  |  |  |  |  |  |  |  |  |  |
| Lao PDR |  |  |  |  |  |  |  |  |  |  |  |  |  |  |  |  |  |
| Lebanon |  |  |  |  |  |  |  |  |  |  |  |  |  |  |  |  |  |
| Liberia |  |  |  |  |  |  |  |  |  |  |  |  |  |  |  |  |  |
| Libya |  |  |  |  |  |  |  |  |  |  |  |  |  |  |  |  |  |
| Madagascar |  |  |  |  |  |  |  |  |  |  |  |  |  |  |  |  |  |
| Malawi |  |  |  |  |  |  |  |  |  |  |  |  |  |  |  |  |  |
| Mali |  |  |  |  |  |  |  |  |  |  |  |  |  |  |  |  |  |
| Marshall Islands |  |  |  |  |  |  |  |  |  |  |  |  |  |  |  |  |  |
| Mauritania |  |  |  |  |  |  |  |  |  |  |  |  |  |  |  |  |  |
| Micronesia |  |  |  |  |  |  |  |  |  |  |  |  |  |  |  |  |  |
| Mozambique |  |  |  |  |  |  |  |  |  |  |  |  |  |  |  |  |  |
| Myanmar |  |  |  |  |  |  |  |  |  |  |  |  |  |  |  |  |  |
| Nepal |  |  |  |  |  |  |  |  |  |  |  |  |  |  |  |  |  |
| Niger |  |  |  |  |  |  |  |  |  |  |  |  |  |  |  |  |  |
| Nigeria |  |  |  |  |  |  |  |  |  |  |  |  |  |  |  |  |  |
| Papua New Guinea |  |  |  |  |  |  |  |  |  |  |  |  |  |  |  |  |  |
| Sao Tome and Principe |  |  |  |  |  |  |  |  |  |  |  |  |  |  |  |  |  |
| Sierra Leone |  |  |  |  |  |  |  |  |  |  |  |  |  |  |  |  |  |
| Solomon Islands |  |  |  |  |  |  |  |  |  |  |  |  |  |  |  |  |  |
| Somalia |  |  |  |  |  |  |  |  |  |  |  |  |  |  |  |  |  |
| South Sudan |  |  |  |  |  |  |  |  |  |  |  |  |  |  |  |  |  |
| Sudan |  |  |  |  |  |  |  |  |  |  |  |  |  |  |  |  |  |
| Syrian Arab Republic |  |  |  |  |  |  |  |  |  |  |  |  |  |  |  |  |  |
| Tajikistan |  |  |  |  |  |  |  |  |  |  |  |  |  |  |  |  |  |
| Timor-Leste |  |  |  |  |  |  |  |  |  |  |  |  |  |  |  |  |  |
| Togo |  |  |  |  |  |  |  |  |  |  |  |  |  |  |  |  |  |
| Tonga |  |  |  |  |  |  |  |  |  |  |  |  |  |  |  |  |  |
| Tuvalu |  |  |  |  |  |  |  |  |  |  |  |  |  |  |  |  |  |
| Uzbekistan |  |  |  |  |  |  |  |  |  |  |  |  |  |  |  |  |  |
| Vanuatu |  |  |  |  |  |  |  |  |  |  |  |  |  |  |  |  |  |
| Venezuela |  |  |  |  |  |  |  |  |  |  |  |  |  |  |  |  |  |
| West Bank and Gaza |  |  |  |  |  |  |  |  |  |  |  |  |  |  |  |  |  |
| Western Sahara |  |  |  |  |  |  |  |  |  |  |  |  |  |  |  |  |  |
| Yemen |  |  |  |  |  |  |  |  |  |  |  |  |  |  |  |  |  |
| Zimbabwe |  |  |  |  |  |  |  |  |  |  |  |  |  |  |  |  |  |

Note: It was not possible to further differentiate among high-intensity conflict, medium-intensity conflict, and high institutional and social fragility, as these nuances were formally introduced in 2020, and the retrospective attribution of a context to a specific category would have been arbitrary and not based on a comprehensive methodology such as the one adopted by the World Bank. Studies from these countries were included if they were conducted during the time in which the country was formally classified as FCAS, or within the following 5 years, which were assumed to be the spill over time in terms of health system disruption and recovery. The same cut-off was also used in similar studies analysing health care services delivery in conflict-affected settings^[[1]](#footnote-2),^^[[2]](#footnote-3),^^[[3]](#footnote-4),^^[[4]](#footnote-5)^.

## Appendix III: Search strategy

### III.a Search strategy adopted in PubMed

| **Item** | **Search strings** |
| --- | --- |
| #1 | ("Health Services"[Mesh] OR "Health Services Research"[Mesh] OR "Health Services Administration"[Mesh] OR "Health Policy"[Mesh] OR "Delivery of Health Care"[Mesh] OR "Health Planning"[Mesh] OR "Health Planning Organizations"[Mesh] OR "Health Workforce"[Mesh]) |
| #2 | ("health system*"[Title/Abstract] OR "health service*"[Title/Abstract] OR "health polic*"[Title/Abstract] OR "health care"[Title/Abstract] OR "healthcare"[Title/Abstract] OR "health administrat*"[Title/Abstract] OR "health financ*"[Title/Abstract] OR "health plan*"[Title/Abstract] "health plan*"[Title/Abstract]) |
| #3 | #1 OR #2 |
| #4 | **("High Reliability Organizations"[Mesh] OR "Models, Organizational"[Mesh] OR "Efficiency, Organizational"[Mesh])** |
| #5 | **(resilien*[Title/Abstract] OR "organization* model*"[Title/Abstract] OR "organization* efficien*"[Title/Abstract])** |
| #6 | **#4 OR #5** |
| #7 | **("Armed Conflicts"[Mesh] OR "Iraq War, 2003-2011"[Mesh] OR "War Exposure"[Mesh] OR "Ethnic Violence"[Mesh] OR "Afghan Campaign 2001-"[Mesh] OR "Violence"[Mesh] OR "Gun Violence"[Mesh] OR "Civil Disorders"[Mesh] OR "Disasters"[Mesh] OR "Disaster Planning"[Mesh] OR "Mass Casualty Incidents"[Mesh] OR "Refugee Camps"[Mesh] OR "Rescue Work"[Mesh] OR "Relief Work"[Mesh] OR "Altruism"[Mesh] OR "Faith-Based Organizations"[Mesh] OR "Organizations, Nonprofit"[Mesh] OR "United Nations"[Mesh] OR "Red Cross"[Mesh])** |
| #8 | **("armed conflict*"[Title/Abstract] OR "conflict-affected"[Title/Abstract] OR war[Title/Abstract] OR violen*[Title/Abstract] OR disaster*[Title/Abstract] OR crisis[Title/Abstract] OR crises[Title/Abstract] OR "mass casualt*"[Title/Abstract] OR refugee*[Title/Abstract] OR humanitarian[Title/Abstract] OR rescue*[Title/Abstract] OR relief[Title/Abstract] OR "non-governmental organization*"[Title/Abstract] OR "faith-based organization*"[Title/Abstract] OR "international organization*"[Title/Abstract] OR "united nation*"[Title/Abstract] OR "red cross"[Title/Abstract] OR "red crescent"[Title/Abstract] OR "red crystal"[Title/Abstract] OR "red crystal"[Title/Abstract] OR ICRC[Title/Abstract] OR IFRC[Title/Abstract]) OR fragil* OR afghan*[Title/Abstract] OR angol*[Title/Abstract] OR armenia*[Title/Abstract] OR azerbaijan*[Title/Abstract] OR bosnia*[Title/Abstract] OR herzegovina[Title/Abstract] OR "burkina faso"[Title/Abstract] OR burundi[Title/Abstract] OR cambodia[Title/Abstract] OR cameroon*[Title/Abstract] OR "central african republic"[Title/Abstract] OR chad[Title/Abstract] OR comoros[Title/Abstract] OR congo*[Title/Abstract] OR "côte d'ivoire"[Title/Abstract] OR djibouti[Title/Abstract] OR eritrea*[Title/Abstract] OR ethiopia*[Title/Abstract] OR gambia[Title/Abstract] OR georgia*[Title/Abstract] OR guinea*[Title/Abstract] OR haiti*[Title/Abstract] OR iraq*[Title/Abstract] OR kiribati[Title/Abstract] OR kosov*[Title/Abstract] OR lao[Title/Abstract] OR leban*[Title/Abstract] OR liberia*[Title/Abstract] OR libya*[Title/Abstract] OR madagascar[Title/Abstract] OR malawi*[Title/Abstract] OR mali[Title/Abstract] OR "marshall island"[Title/Abstract] OR mauritania*[Title/Abstract] OR micronesia*[Title/Abstract] OR mozambi*[Title/Abstract] OR myanmar[Title/Abstract] OR burma[Title/Abstract] OR nepal*[Title/Abstract] OR niger*[Title/Abstract] OR "papua new guinea"[Title/Abstract] OR "sao tome and principe"[Title/Abstract] OR "sierra leone"[Title/Abstract] OR "solomon island"[Title/Abstract] OR somali*[Title/Abstract] OR sudan*[Title/Abstract] OR syria*[Title/Abstract] OR tajikistan[Title/Abstract] OR "timor-leste"[Title/Abstract] OR togo[Title/Abstract] OR tonga[Title/Abstract] OR tuvalu[Title/Abstract] OR uzbekistan[Title/Abstract] OR vanuatu[Title/Abstract] OR venezuela[Title/Abstract] OR palestin*[Title/Abstract] OR "west bank"[Title/Abstract] OR gaza[Title/Abstract] OR "western sahara"[Title/Abstract] OR yemen*[Title/Abstract] OR zimbabw*[Title/Abstract]** |
| #9 | **#7 OR #8** |
| #10 | **#3 AND #6 AND #9** |
| #11 | Filters: **from 2006/1/1 - 2022/2/21** |
| #12 | #10 AND #11 |
| #13 | Filters: Arabic, **English, French, German, Italian, Portuguese, Spanish** |
| #14 | #12 AND #13 |

### III.b Search strategy adopted in Scopus

| **Item** | **Search strings** |
| --- | --- |
| #1 | TITLE-ABS-KEY ( "health system*" OR "health service*" OR "health polic*" OR "health care" OR "healthcare" OR "health administrat*" OR "health financ*" OR "health plan*" ) |
| #2 | TITLE-ABS-KEY ( resilien* OR "organization* model*" OR "organization* efficien*" ) |
| #3 | TITLE-ABS-KEY ( "armed conflict*" OR "conflict-affected" OR war OR violen* OR disaster* OR crisis OR crises OR "mass casualt*" OR refugee* OR humanitarian OR rescue* OR relief OR "non-governmental organization*" OR "faith-based organization*" OR "international organization*" OR "united nation*" OR "red cross" OR "red crescent" OR "red crystal" OR icrc OR ifrc OR fragil* OR fghan* OR angol* OR armenia* OR azerbaijan* OR bosnia* OR herzegovina OR "burkina faso" OR burundi OR cambodia OR cameroon* OR "central african republic" OR chad OR comoros OR congo* OR "côte d'ivoire" OR djibouti OR eritrea* OR ethiopia* OR gambia OR georgia* OR guinea* OR haiti* OR iraq* OR kiribati OR kosov* OR lao OR leban* OR liberia* OR libya* OR madagascar OR malawi* OR mali OR "marshall island" OR mauritania* OR micronesia* OR mozambi* OR myanmar OR burma OR nepal* OR niger* OR "papua new guinea" OR "sao tome and principe" OR "sierra leone" OR "solomon island" OR somali* OR sudan* OR syria* OR tajikistan OR "timor-leste" OR togo OR tonga OR tuvalu OR uzbekistan OR vanuatu OR venezuela OR palestin* OR "west bank" OR gaza OR "western sahara" OR yemen* OR zimbabw* ) |
| #4 | **#1 AND #2 AND #3** |
| #5 | PUBYEAR  >  2006 |
| #6 | **#4 AND #5** |
| #7 | **( LIMIT-TO ( LANGUAGE , "English" ) OR LIMIT-TO ( LANGUAGE , "French" ) OR LIMIT-TO ( LANGUAGE , "Spanish" ) OR LIMIT-TO ( LANGUAGE , "German" ) OR LIMIT-TO ( LANGUAGE , "Italian" ) OR LIMIT-TO ( LANGUAGE , "Portuguese" ) )** |
| #8 | **#6 AND 7** |

### III.c Search strategy adopted in Web of Science

| **Item** | **Search strings** |
| --- | --- |
| #1 | "health system*" OR "health service*" OR "health polic*" OR "health care" OR "healthcare" OR "health administrat*" OR "health financ*" OR "health plan*" (Title) |
| #2 | "health system*" OR "health service*" OR "health polic*" OR "health care" OR "healthcare" OR "health administrat*" OR "health financ*" OR "health plan*" (Abstract) |
| #3 | #1 OR #2 |
| #4 | **resilien* OR "organization* model*" OR "organization* efficien*" (Title)** |
| #5 | **resilien* OR "organization* model*" OR "organization* efficien*" (Abstract)** |
| #6 | **#4 OR #5** |
| #7 | **"armed conflict*" OR "conflict-affected" OR war OR violen* OR disaster* OR crisis OR crises OR "mass casualt*" OR refugee* OR humanitarian OR rescue* OR relief OR "non-governmental organization*" OR "faith-based organization*" OR "international organization*" OR "united nation*" OR "red cross" OR "red crescent" OR "red crystal" OR icrc OR ifrc OR fragil* OR fghan* OR angol* OR armenia* OR azerbaijan* OR bosnia* OR herzegovina OR "burkina faso" OR burundi OR cambodia OR cameroon* OR "central african republic" OR chad OR comoros OR congo* OR "côte d'ivoire" OR djibouti OR eritrea* OR ethiopia* OR gambia OR georgia* OR guinea* OR haiti* OR iraq* OR kiribati OR kosov* OR lao OR leban* OR liberia* OR libya* OR madagascar OR malawi* OR mali OR "marshall island" OR mauritania* OR micronesia* OR mozambi* OR myanmar OR burma OR nepal* OR niger* OR "papua new guinea" OR "sao tome and principe" OR "sierra leone" OR "solomon island" OR somali* OR sudan* OR syria* OR tajikistan OR "timor-leste" OR togo OR tonga OR tuvalu OR uzbekistan OR vanuatu OR venezuela OR palestin* OR "west bank" OR gaza OR "western sahara" OR yemen* OR zimbabw* (Title)** |
| #8 | **"armed conflict*" OR "conflict-affected" OR war OR violen* OR disaster* OR crisis OR crises OR "mass casualt*" OR refugee* OR humanitarian OR rescue* OR relief OR "non-governmental organization*" OR "faith-based organization*" OR "international organization*" OR "united nation*" OR "red cross" OR "red crescent" OR "red crystal" OR icrc OR ifrc OR fragil* OR fghan* OR angol* OR armenia* OR azerbaijan* OR bosnia* OR herzegovina OR "burkina faso" OR burundi OR cambodia OR cameroon* OR "central african republic" OR chad OR comoros OR congo* OR "côte d'ivoire" OR djibouti OR eritrea* OR ethiopia* OR gambia OR georgia* OR guinea* OR haiti* OR iraq* OR kiribati OR kosov* OR lao OR leban* OR liberia* OR libya* OR madagascar OR malawi* OR mali OR "marshall island" OR mauritania* OR micronesia* OR mozambi* OR myanmar OR burma OR nepal* OR niger* OR "papua new guinea" OR "sao tome and principe" OR "sierra leone" OR "solomon island" OR somali* OR sudan* OR syria* OR tajikistan OR "timor-leste" OR togo OR tonga OR tuvalu OR uzbekistan OR vanuatu OR venezuela OR palestin* OR "west bank" OR gaza OR "western sahara" OR yemen* OR zimbabw* (Abstract)** |
| #9 | **#7 OR #8** |
| #10 | **#3 AND #6 AND #9** |
| #11 | **2006-01-01 to 2022-02-21 (Publication Date)** |
| #12 | #10 AND #11 |
| #13 | Arabic OR **English OR French OR German OR Italian OR Portuguese OR Spanish** (Languages) |
| #14 | #12 AND #13 |

## Appendix IV: Data extraction tool

| **Thematic group** | **Description** |
| --- | --- |
| **Study Description** | 1. First author and year of publication 2. DOI 3. Study title 4. Study period 5. Study objective(s) 6. Study methodology 7. Health domain of focus 8. Level of care 9. Working definition of health system resilience 10. Resilience framework applied |
| **Context of resilience** | Geographical setting and contextual conditions |
| **Phenomena of resilience** | 1. Goals/objectives of resilience (i.e. *resilience for what?*) 2. Triggers of resilience (i.e. *resilience to what?*) 3. Materials of resilience (i.e. *resilience of what?*) 4. Mechanisms of resilience (i.e. *resilience through what?*) |
| **Emerging themes** | Themes not included in existing frameworks emerging from the study |

## Appendix V: Summary of the characteristics of the 37 articles and reports included

| **First author** | **Year** | **Title** | **Context** | **Health domain(s)** | **Level(s) of care** | **Objective(s) of the study** | **Framework adopted** | **Shock(s) to the health system** | **Goal(s) of resilience** | **Summary** |
| --- | --- | --- | --- | --- | --- | --- | --- | --- | --- | --- |
| Lancet Editors | 2014 | *The silver bullet of resilience* | West African countries affected by the 2014-2016 Ebola outbreak | Communicable diseases control | Governance | N/A | Not specified | Infectious disease outbreak (Ebola) | Not defined | The editorial analyses briefly the delays in the international response to the Ebola outbreak, while providing suggestions on how to build resilient health systems through a concerted action that sees involved different actors, from governments to international organizations and private companies. |
| Ager et al. | 2015 | *Health service resilience in Yobe state, Nigeria in the context of the Boko Haram insurgency: a systems dynamics analysis using group model building* | Yobe State, Northern Nigeria, following the Boko Haram upraising from 2011 to 2013. | Maternal, Newborn and Child Care | Primary care | To identify key pathways of response and adaptation to the threats that an armed group insurgency provoked for the provision of primary health care services through system dynamics analysis | Kruk’s characteristics of resilient health systems | Armed conflict and violence (armed group violence) | Continuity of care provision | The study identifies several main threats to continuity of primary health care provision following an armed group insurgency in the North of Nigeria. These include: insecurity, which jeopardized access to care for the population and freedom of movements of health care workers; migration of health care workers and excessive workload for those remaining; disruptions in the supply chain for medicines and equipment; and financial barriers.  Some of the main pathways of adaptation that generated resilience for service provision included coordination between health care system and security services; task shifting in the health care workforce; financial support; staff commitment and motivation; and community cohesion. |
| Kieny et al. | 2015 | *Beyond Ebola: a new agenda for resilient health systems* | West African countries affected by the 2014-2016 Ebola outbreak from the perspective of a high-level meeting held  in Geneva in December 2014, with the participation of Ministers of Health and Finance of the affected countries, international organisations, and development actors | Communicable diseases control | Governance | N/A | Not specified | Infectious disease outbreak (Ebola) | Continuity of care provision | The commentary discusses the proposed strategies to strengthen health systems resilience in the wake of infectious diseases outbreaks. Among these there are:(Ager et al., 2015; Kieny & Dovlo, 2015) integrated inter-sectoral approaches, substantial external financial support to Ebola-affected countries, better coordination, early warning systems, community engagement, expansion of health care workforce, and increased accountability of local governments and international actors. |
| Kruk et al. | 2015 | *What is a resilient health system? Lessons from Ebola* | West African countries affected by the 2014-2016 Ebola outbreak | Communicable diseases control | Governance | N/A | Kruk’s characteristics of resilient health systems | Infectious disease outbreak (Ebola) | Health security | The viewpoint brings a reflection on the main lessons learned following the onset of the Ebola outbreak in West Africa for health systems resilience. These include the need for improved accountability at all levels of the response to global health threats, strengthening and supporting the health care workforce, and acknowledging the key role of social capital within affected communities. It concludes with proposing a new framework for health systems resilience defining five characterizing elements: awareness, diversity, self-regulation, integration, and adaptivity, providing a contextualized description for each of them. |
| Martineau | 2016 | *People-centred health systems: building more resilient health systems in the wake of the Ebola crisis* | West African countries affected by the 2014-2016 Ebola outbreak | Communicable diseases control | Governance | N/A | Not specified | Infectious disease outbreak (Ebola) | Not specified | The commentary describes the shortcomings of the global response to the Ebola outbreak, emphasizing on the need for solutions that aim at empowering local communities and strengthen local systems. It advocates for a “people-centred” approach that supports trust building among the different interconnected actors involved in health systems in fragile contexts. |
| McKenzie et al. | 2016 | *Creating the Foundation for Health System Resilience in Northern Nigeria* | Four states in Northern Nigeria: Jigawa, Katsina, Yobe, and Zamfara | Maternal, Newborn and Child Care | Primary | To describe the Partnership for Reviving Routine  Immunization in Northern Nigeria: Maternal Newborn  and Child Health (PRRINN-MNCH) program, and draw from it lessons learned for strengthening health systems resilience | Kruk’s characteristics of resilient health systems | Armed conflict and violence (armed group violence) | Continuity of care provision | The article describes the PRRINN-MNCH program delivered in Norther Nigeria and how it evolved following the Boko Haram insurgency adopting a complex adaptive system theory of change approach. It describes in particular the operational strategies that have promoted resilience for continuity of provision of quality primary care services in the wake of major shocks to the primary health care system provoked by the increasing violence triggered by the armed group in the intervention areas. In particular, it highlights how decentralization promotes a meaningful engagement of local stakeholders |
| Kruk et al. | 2017 | *Building resilient health systems: a proposal for a resilience index* | For the purpose of this review, the following two settings were the focus of the analysis: Lebanon and Liberia | Not specified | Primary, Governance | To draw a resilience index from the presented case studies of Lebanon and Liberia | Kruk’s characteristics of resilient health systems | Armed conflict and violence (influx of refugees in Lebanon); infectious disease outbreak (Ebola in Liberia) | Health security | This analysis paper builds on the five characteristics of resilient health systems (awareness, self-regulation, adaptivity, integration, and diversity), applying this lens to specific case studies of Lebanon, Liberia and Indonesia. For the purpose of this review, only the first two are included. The adaptations of the primary health care system in Lebanon is described as an example of integration of the diversity of care providers in the countries. The case of Liberia is offered instead as an example of self-regulation in the adaptations of service provision and communication strategies in the wake of the Ebola outbreak. From these examples, a resilience index is proposed as a way to measure resilience. |
| Ling et al. | 2017 | *Beyond the crisis: did the Ebola epidemic improve resilience of Liberia’s health system?* | Liberia in the wake of the 2014-2016 Ebola outbreak | Communicable diseases control | Governance | To describe local, national and global stakeholders’ priorities for health system resilience in Liberia through the lens of the five-characteristic resilience framework | Kruk’s characteristics of resilient health systems | Infectious disease outbreak (Ebola) | Health security | The paper reports the findings of a qualitative study exploring the priorities for the Liberia health system’s resilience from the perspective of different actors involved in the response to the Ebola outbreak. It posits that the advancements observed in Liberia were mainly reported in the elements of resilience prioritized by global actors (i.e. integration, awareness and adaptivity) rather than those prioritized by local actors (i.e. self-regulation and diversity). It concludes stressing the importance of including local stakeholders’ perspectives and priorities in interventions oriented at strengthening health system resilience. |
| Mangouri et al. | 2017 | *Exploring resilience in a complex crisis: Iraq's health system response to a triple security, financial and ecological shock* | Iraq | Not specified | Governance | To identify barriers and enablers for continuity of service provision in conflict affected areas of Iraq between 2014 and 2015 | Not specified | Armed conflict and violence; financial crisis; environmental crisis | Continuity of care provision | The conference abstract summarizes the findings of a qualitative study conducted in Iraq. It identifies lack of financial resources as the main barrier for health system resilience, together with shortages of health care workers and supplies, and weak information management systems. On the other hand, the enablers of resilience appear to be staff motivation and commitment, humanitarian pooled funding, and external support provided by international organization. |
| Martineau et al. | 2017 | *Leaving no one behind: lessons on rebuilding health systems in conflict- and crisis-affected states* | For the purpose of this review, the following three settings were the focus of the analysis: Cambodia, Sierra Leone and Zimbabwe | Not specified | Governance | To summarize lessons learned from a research partnership (ReBUILD consortium) on how it supported health systems resilience strengthening in fragile and conflict-affected settings | ReBUILD | Armed conflict and violence | Universal Health Coverage | The analysis paper describes three overarching themes that have emerged from the ReBUILD consortium experience as key players in health systems resilience. The first element is the need for meaningful inclusion of affected communities in program design; the second one the imperative of strengthening the health care workforce for building health systems resilience; and the third one to support local institutions being mindful of power imbalances when external aid actors come in. |
| Nyenswah | 2017 | *Reflections on Leadership and Governance from the Incident Manager of Liberia's Ebola Response* | Liberia during the 2014-2016 Ebola outbreak | Communicable diseases control | Governance | To summarize the lessons learned from the management of the Ebola outbreak response from the point of view of the incident manager | Not specified | Infectious disease outbreak (Ebola) | Health security | The opinion piece provides the insight of the incident manager of Liberia’s Ebola response. It summarizes the developments in the Liberia’s Ministry of Health following the epidemic shock, mainly through two major changes in the stewardship of the health system. The first one was the Joint External Evaluation led by the World Health Organization, which allowed to identify strengths to capitalize on and weaknesses to mitigate. The second one was the establishment of a Liberian Public Health Institute with the overarching goal of providing high-level technical assistance to the government to enhance resilience of the health care system against infectious diseases hazards. |
| Peters | 2017 | *The Ebola epidemic in Liberia: the role of communities and local leadership in overcoming catastrophe and building health system resilience* | West African countries affected by the 2014-2016 Ebola outbreak | Communicable diseases control | Governance | N/A | Not specified | Infectious disease outbreak (Ebola) | Not specified | The conference abstract highlights the delays in the international response to the Ebola outbreak that hit West Africa on one side, and the critical contribution of local communities and institutions in controlling instead the epidemic on the other one. |
| Sochas et al. | 2017 | *Counting indirect crisis-related deaths in the context of a low-resilience health system: the case of maternal and neonatal health during the Ebola epidemic in Sierra Leone* | Sierra Leone during the 2014-2016 Ebola outbreak | Maternal, Newborn and Child Care | Primary | To pilot test a rapid assessment method to evaluate indirect mortality (maternal, neonatal deaths and stillbirth) during the Ebola outbreak | Not specified | Infectious disease outbreak (Ebola) | Health security | The study uses routine data collected through the Sierra Leone health management information system inputting them into the Lives Saved Tool, in order to obtain an estimate of indirect mortality in maternal, newborn and child care, due to the disruption of essential services provoked by the Ebola outbreak. It concludes that the burden of indirect deaths is at least of the same magnitude of the burden of direct mortality due to the outbreak in a non-resilient health system. It concludes stressing the importance of adequate data flow to inform prioritization of interventions in resource-limited settings. |
| van de Pas et al. | 2017 | *Interrogating resilience in health systems development* | Gaza in the Occupied Palestinian Territories | Not specified | Governance | To describe the outcome of a panel discussion at the roundtable on ‘Resilient and responsive  health systems for a changing world’ during the fourth Global Symposium on Health Systems Research | Not specified | Armed conflict and violence | Universal Health Coverage | The paper provides a critique of the concept of resilience, arguing that, in the current narrative, this is mainly supporting an agenda driven by international organizations in the Global North. As such, it aims at maintaining structural inequities in low- and middle-income countries, which often bear a disproportionate burden of fragility and conflict. This critique is exemplified by the case study of Gaza, where decades of international interventions in support to the health system have not contributed to a solution of the structural vulnerabilities affecting the population living in this context. |
| Witter et al. | 2017 | *How do health workers experience and cope with shocks? Learning from four fragile and conflict-affected health systems in Uganda, Sierra Leone, Zimbabwe and Cambodia* | For the purpose of this review, the following three settings were the focus of the analysis: Cambodia, Sierra Leone and Zimbabwe | Not specified | Governance | To summarize lessons learned from a research partnership (ReBUILD consortium) on how to support health care workforce | Not specified | Armed conflict and violence; infectious disease outbreak (Ebola); economic - political crisis | Universal Health Coverage | The article described the main challenges and coping mechanisms of the health care workforce operating in conflict settings, to draw recommendations on how to strengthen the resilience of health staff, which in turn can boost the resilience of the health systems they operate in. The main challenges unfold both at personal and professional level: from being victims of violence to suffer from financial hardship, from increased workload to lack of resources to respond to the emerging needs. The coping mechanisms emerging range from the realm of personal values, commitment and motivation, to external financial incentives. It concludes by highlighting the need to address the gaps in support and protection of health staff in order to strengthen health systems resilience in situation of crises. |
| Atallah et al. | 2018 | *Developing Equitable Primary Health Care in Conflict-Affected Settings: Expert Perspectives From the Frontlines* | Fragile and Conflict-Affected Settings in general | Not specified | Primary | To understand how to build equitable primary health care in conflict-affected settings building on the views of global experts | New framework proposed: five building blocks, three intermediating factors and a four-element roadmap | Armed conflict and violence | Universal Health Coverage | Through a grounded theory analysis of semi-structured interviews with global expert, this study explores strategies to develop people-centred, equitable PHC services in areas affected by structural violence. The emerging framework is articulated around building blocks (i.e. contextual elements and the modalities to analyse them), intermediating factors (i.e. factors affecting the building blocks), and an operational roadmap (focused on communities and specific interventions to serve them). |
| Hanefeld et al. | 2018 | *Towards an understanding of resilience: responding to health systems shocks* | For the purpose of this review, the focus of the analysis in on West African countries affected by the 2014-2016 Ebola outbreak | Communicable diseases control | Governance | To analyse different types of health systems response to shocks to derive a proposal for a new analytical framework of interventions aiming at strengthening health systems resilience. | New framework proposed: “3 plus 2” dimensions of health systems resilience (health information management system, funding, health workforce, plus governance and values/belief) | Infectious disease outbreak (Ebola) | Not specified | Based on the analysis of different types of shocks to health systems, the authors propose a framework for health systems resilience based on three core dimensions (i.e. health information systems, funding, and health care workforce) and two cross-cutting elements (i.e. governance and values). |
| Miller et al. | 2018 | *Community health workers during the Ebola outbreak in Guinea, Liberia, and Sierra Leone* | West African countries affected by the 2014-2016 Ebola outbreak | Maternal, Newborn and Child Care | Primary | To describe the role of community health workers (CHW) in the Ebola response, and to explore how their work contributed to health systems resilience | iCCM (integrated Community Case Management of childhood illness) benchmarks framework | Infectious disease outbreak (Ebola) | Continuity of service provision | The paper summarises a mixed-method study exploring the experience of CHW in providing routine maternal, newoborn and child health (MNCH) services during the Ebola outbreak in West Africa. Despite the disruptions in provision of MNCH services, CHW maintained a presence in their communities and supported some continuity of service provision. It concludes by hypothesizing how a more structured and meaningful support to the CHW’s role could play a role in maintaining better continuity of service provision during a crisis. |
| Alameddinee et al. | 2019 | *Resilience capacities of health systems: Accommodating the needs of Palestinian refugees from Syria* | For the purpose of this review, the focus of the analysis in on Lebanon, affected by the influx of Syrian refugees | Not specified | Primary | To appraise the validity of Blanchet’s capacity-oriented health systems resilience framework in analyzing the UNRWA health system’s response to the influx of Syrian refugees in Lebanon (and in Jordan) | Blanchet’s capacity-oriented framework | Armed conflict and violence | Continuity of service provision | The study provides an analysis of the UNRWA response to population displacements from Syria to Lebanon (and Jordan), and describes its absorptive, adaptive and transformative capacities in providing continuity of care provision for Palestinian refugees coming from Syria. It concludes highlighting the relevance of a capacity-oriented resilience framework in appraising a health system strategy of response to shocks. |
| Alonge et al. | 2019 | *Understanding the role of community resilience in addressing the Ebola virus disease epidemic in Liberia: a qualitative study* | Liberia during the 2014-2016 Ebola outbreak | Communicable diseases control | Primary | To understand the role that community resilience played in supporting the resilience of the Liberia health system during the Ebola outbreak | Blanchet’s capacity-oriented framework | Infectious disease outbreak (Ebola) | Health security | The study identifies the key enablers of community resilience in relation to their contribution to the resilience of the Liberian health care system during the Ebola outbreak: these are strong leadership; social cohesion within the community; the use of trusted communication channels; and transversal trust among different health actors. It concludes by highlighting how community resilience can play a significant role in ensuring resilience of the broader health systems during shocks. |
| Drevin et al. | 2019 | *”For this one, let me take the risk”: why surgical staff continued to perform caesarean sections during the 2014–2016 Ebola epidemic in Sierra Leone* | Sierra Leone during the 2014-2016 Ebola outbreak | Maternal, Newborn and Child Care | Secondary | To understand the perspective and drivers of motivation of Sierra Leone health care workers who continued to offer cesarean sections during the Ebola outbreak | WHO building blocks | Infectious disease outbreak (Ebola) | Continuity of service provision | The study identifies the following drivers of continuity of cesarean section provision in public hospitals in Sierra Leone during the Ebola outbreak: 1) the intrinsic motivation and commitment of health care workers involved in emergency obstetric surgery provision; and 2) the flexibility and creativity in adapting clinical practices to the emerging risks. |
| Tjoflåt et al. | 2019 | *Building Resilience in Humanitarian Hospital Programs During Protracted Conflicts: Opportunities and Limitations* | Fragile and Conflict-Affected Settings in general | Trauma care | Secondary | N/A | Not specified | Armed conflict and violence | Continuity of care provision | The book chapter is based on the field experience of the authors and discusses how humanitarian organizations supporting hospital care provision in conflict affected settings can support the resilience of the system in which these hospitals operate. The central elements suggested are: 1) a partnership approach; 2) a knowledge sharing system; and 3) material support in terms of equipment and supplies. |
| Bang et al. | 2020 | *Gauging Cameroon’s resilience to the COVID-19 pandemic: implications for enduring a novel health crisis* | Cameroon at the onset of the COVID-19 pandemic | Communicable diseases control | Governance | To appraise the resilience of Cameroon’s health system in its response at the onset of the COVID-19 pandemic | Health System Resilience for Emerging Infectious Diseases (HSREID) framework | Infectious disease outbreak (COVID-19) | Universal Health Coverage | The study applies the Health Systems Resilience for Emerging Infectious Diseases (HSREID) framework to the Cameroon’s health system early response to COVID-19. It identifies major shortcomings in all hardware (infrastructure, supplies, health workforce, and coordination) components, as well as in the software components (governance and trust). It concludes with the observation that the system lacked resilience, identifying key areas for future improvements. |
| Barker et al. | 2020 | *Community engagement for health system resilience: evidence from Liberia’s Ebola epidemic* | Liberia during the 2014-2016 Ebola outbreak | Communicable diseases control | Governance | To analyse the contribution of community engagement for the resilience of the Liberian health system in response to the Ebola outbreak | Kruk’s characteristics of resilient health systems | Infectious disease outbreak (Ebola) | Health security | The study applies Kruk’s framework of the five characteristic of resilient health systems to analyse the role of community engagement in the Liberian Ebola response. It identifies four main approaches: information provision, consultation, participation, and community empowerment. It concludes by highlighting how such strategies can support trust building and hence increase effectiveness of the health responses during crises. |
| Bou-Karroum et al. ^[[5]](#footnote-6)^ | 2020 | *Building a resilient health workforce in fragile and conflict-affected countries to respond to the COVID-19 pandemic and beyond* | Fragile and Conflict-Affected Settings in general | Communicable diseases control | Governance | N/A | Not specified | Infectious disease outbreak (COVID-19) | Continuity of care provision | The policy report begins by analysing the main challenges faced by health care workers during the COVID pandemic, identified in structural deficiencies (depletion of health care workers and limited resources), inadequate trainings, deliberate violence and increased workload causing mental health concerns, limited financial protection, and lack of informative data on their health outcomes. It concludes suggesting operational strategies to address each the identified challenges. |
| Dean et al. | 2020 | *Psychological resilience, fragility and the health workforce: lessons on pandemic preparedness from Liberia and Sierra Leone* | Liberia during the 2014-2016 Ebola outbreak | Not specified | Governance | N/A | Not specified | Infectious disease outbreak (Ebola) | Continuity of care provision | The commentary explores the interconnectedness between the resilience of health care workers and the resilience of the health system as a whole, drawing on key lessons learned during the Ebola outbreak in Sierra Leone and Liberia. Among these, providing psycho-social support interventions to frontline health workers; addressing resource constraints that make workload overwhelming; and promoting social cohesion by addressing the stigma often faced by health workers. |
| Jamal et al. | 2020 | *Health system resilience in the face of crisis: analysing the challenges, strategies and capacities for UNRWA in Syria* | Syria after the onset of the armed conflict in 2011 | Not specified | Primary | To analyse the resilience of UNRWA health system in ensuring continuity of service provision for Palestinian refugees in Syria through the capacity-oriented resilience framework proposed by Blanchet | Blanchet’s capacity-oriented framework | Armed conflict and violence | Continuity of care provision | The study summarizes the strategies that UNRWA has adopted to ensure continuity of care provision for Palestinian refugees in Syria after the onset of the armed conflict in 2011. The absorptive strategies emerging are mainly related to adjustments in the workforce and resources available to ensure flexible response to the emerging needs. Adaptive strategies included a lean supply chain management and decentralized decision making to promote efficiency and effectiveness of the response. Transformative strategies encompassed the expansion of service portfolio to respond to emerging needs both among the affected population, and within the health care workforce. |
| Odhiambo et al. | 2020 | *Measuring health system resilience in a highly fragile nation during protracted conflict: South Sudan 2011–15* | 9 out of the 10 States of South Sudan during the 2011-2015 period of the conflict | MNCH | Primary | To test the application of a constructed resilience index to different definitions of resilience, in order to understand how to measure resilience in FCAS | Not specified | Armed conflict and violence | Continuity of care provision | The authors propose a resilience index, built with MNCH coverage indicators and from conflict-related, stress indicators. The index is applied to three different definitions of health systems resilience (maintaining function, improving function, and achieving national targets). The findings show that the level of stressed produced by the conflict is not necessarily associated with lack of resilience, by virtue of other factors coming into play (such as redundancies in the system, external aid, etc.). |
| Altare et al. | 2021 | *From Insecurity to Health Service Delivery: Pathways and System Response Strategies in the Eastern Democratic Republic of the Congo* | North and South Kivu provinces in the Democratic Republic of Congo | MNCH | Primary and secondary | To describe the resilience of the North and South Kivu health systems in response to chronic levels of violence | Blanchet’s capacity-oriented framework | Armed conflict and violence | Universal Health Coverage | The study identifies three key pathways through which the chronic insecurity affecting the study context impact health service provision and quality: 1) reduced mobility and access; 2) violence; and 3) decreased financial resources. The strategies proposed to address the identified challenges are proposed as absorptive, adaptive or transformative interventions, and develop around: a) expanding the healthcare workforce; b) ensuring contingency planning for movements; c) improving safety and acceptance of communities. |
| Christensen et al. | 2021 | *Building Resilient Health Systems: Experimental Evidence from Sierra Leone and the 2014 Ebola Outbreak* | Four districts of Sierra Leone during the 2014-2016 Ebola outbreak | MNCH | Primary | To understand whether the health system strengthening interventions delivered prior to the Ebola outbreak onset in Sierra Leone have contributed to the resilience of its health system in dealing with the epidemic | Not specified | Infectious disease outbreak (Ebola) | Health security | The study analyses two different strategies of intervention to support quality of care: community monitoring, and non-financial incentives for health staff. It investigates, through the analysis of routine data, how these interventions, started before the onset of Ebola, affected the resilience of MNCH services during the Ebola outbreak. It shows that both types of interventions supported the resilience of the local health system, as they led to a reduction in under 5 mortality among children, as well as an increase in reporting of Ebola cases. |
| Cristiano et al. | 2021 | *Systemic sustainability and resilience assessment of health systems, addressing global societal priorities: Learnings from a top nonprofit hospital in a bioclimatic building in Africa* | A tertiary level hospital providing specialized cardiac surgery in Khartoum, capital of Sudan | Specialised surgical care | Tertiary | To test emergy accounting and life-cycle assessment as analytical tools to understand the energy performance of a hospital and requirements for the system in which it operates, characterized by adverse climatic conditions | Not specified | Climate-related stress | Continuity of care provision | The paper presents a case study of a tertiary hospital, looking at how its climate resilience can strengthen the resilience of the health system in which it operates. It applies an emergy accounting approach along with life-cycle assessment evaluation, showing how specific consideration in the architectural and engineering design of a health facility can decrease its environmental impact, and hence strengthen the sustainability of its operations within a broader health system stressed by several challenges (including not only climatic, but also political, societal and economic triggers of instability). |
| Grimm et al. | 2021 | *Evidence of health system resilience in Myanmar during Cyclone Nargis: a qualitative analysis* | Myanmar after Cyclone Nargis in 2008 | Not specified | Governance | To describe how the Myanmar health system responded to cyclone Nargis | New framework proposed expanding on Kruk’s characteristics of resilient health systems, and placing social capital at its core | Natural disaster (cyclone) | Continuity of care provision | The paper presents the finding of a retrospective qualitative case study, adopting a framework of health systems resilience developed by the authors. It shows that the main drivers of health system resilience during the natural disaster in Myanmar lay in the strong social capital of the country, as well as in the cultural and religious motivation of its communities. It discussed the relevance of capitalizing on such drivers to promote trust and accountability, and hence future resilience. |
| Hamadeh et al. | 2021 | *Working short and working long: can primary healthcare be protected as a public good in Lebanon today?* | Lebanon | Not specified | Primary | N/A | Not specified | Armed conflict and violence (influx of refugees from Syria); infectious disease outbreak (COVID-19); economic - political crisis; man-made disaster (explosion) | Universal Health Coverage | The commentary describes the shocks that the Lebanese Primary Health Care Network has undergone historically, along with the compounding effects of more recent events such as the COVID-19 pandemic, the socio-economic crisis., and the Beirut port explosion. It emphasizes on the adaptations and resilience of the system, advocating for international support to maintain PHC as a necessary safeguard for the most vulnerable populations. |
| Ibrahim et al. | 2021 | *Resilience of health systems in conflict affected governorates of Iraq, 2014–2018* | Four conflict-affected governorates of Iraq from 2014 to 2018 | Not specified | Primary | To describe the resilience of the primary health care system in four conflict-affected governorates of Iraq | Blanchet’s capacity-oriented framework | Armed conflict and violence | Continuity of care provision | The study describes the different strategies adopted by the health system in Iraq to respond to the armed conflict triggered by the ISIS invasion. It describes absorptive, adaptive and transformative operations adopted, highlighting however the lack of preparedness of the system, and the additional challenges in reconstruction created by the loss of trust from the community towards health institution due to the inefficient response. It concludes by providing recommendations on how to address the identified shortcomings. |
| Mayhew et al. | 2021 | *Responding to the 2018-2020 Ebola Virus Outbreak in the Democratic Republic of the Congo: Rethinking Humanitarian Approaches* | The Democratic Republic of Congo during the 2018-2020 Ebola outbreak | Not specified | Governance | To critically analyse how international humanitarian organizations have capitalized on community involvement and trust building to contribute to health system resilience during the Ebola outbreak in DRC | New framework proposed: health systems and social institutions as central element, interacting with people from the local to the international level through either power-based or trust-based relationships | Infectious disease outbreak (Ebola) | Continuity of care provision | The paper reviews the international humanitarian response to the 2018-2020 Ebola outbreak in DRC from a health system resilience perspective. It identifies major shortcomings in the lack of involvement and empowerment of local communities and institution in setting priorities and designing responses, despite the evidence on how critical these elements are in terms of effectiveness of interventions and ultimately trust building. It concludes by advocating for a substantial shift in the approach of international organizations, who need to transform their role within the emergency response system letting go of power and decision making. |
| Sripad et al. | 2021 | *"Eternally restarting" or "a branch line of continuity"? Exploring consequences of external shocks on community health systems in Haiti* | Haiti | Not specified | Primary | To describe how political and environmental shocks have influenced the resilience of community health services in Haitii | Not specified | Natural disaster (earthquake); disease outbreak (cholera); economic - political crisis; | Continuity of care provision | The study analyses the cumulative impact of different types of shocks on the community health system in Haiti. The consequences of these shocks manifest in a complex web of programmatic challenges, such as limitation in resources available, short term cycles of funding, issues with internal governance, establishment of parallel systems. However, despite these challenges, the intrinsic motivation and commitment of community health workers appears to be the backbone for continuity of primary health care service provision, and hence for health system resilience. The authors closure by advocating for formal acknowledgment, also through financial provision, of such critical role. |
| Moitinho de Almeida | 2022 | *“Recovering, not recovered” Hospital disaster resilience: a case-study from the 2015 earthquake in Nepal* | Tertiary hospital in Katmandu, Nepal, in the wake of the earthquake in 2015 | Surgical care | Tertiary | To explore the resilience mechanisms of a tertiary hospital in response to the earthquake of 2015 in Nepal | “4 Rs” resilience framework (two ends of resilience: robustness and rapidity; plus two means of resilience: redundancy and resourcefulness) | Natural disaster (earthquake) | Continuity of service provision | The author presents an in-depth case study of a tertiary hospital, intended as a health system in itself and in relation with the health network it provides for. She describes the robustness of the hospital response in absorbing the increased caseload, as well as the rapidity of the adaptations. She also highlights the redundancies established to allow for adjustments, as well as the pre-existing resources that enabled resiliency (such as the existence of a contingency plan). The resilience of health care workers, sustained mainly by their intrinsic motivation and commitment, is additional discussed as an element emerging from the study, and not possible to include in the 4 R framework applied to the case study. |

## Appendix VI: Absorptive, adaptive and transformative operations for HSR in FCAS

| **Domain** | **Absorptive operations** | **Adaptive operations** | **Transformative operations** |
| --- | --- | --- | --- |
| **Safety and Security** | - - Coordination with security apparatuses to ensure safety of health care workers on one side, and protect access to care for populations on the other *(Ibrahim et al., 2021; Jamal et al., 2020; Odhiambo et al., 2020)*   - Definition of contingency plans *(Altare et al., 2021; Bang et al., 2020;* Moitinho de Almeida, 2022) | - Enhanced coordination with armed and security forces to maintain access to health care *(Ager et al., 2015)* - Adoption of additional measures to protect health care workforce *(Bou-Karroum et al., 2020)* | - Adoption of evidence-based strategy to ensure safety and security of populations, health care workers, health facilities and medical goods *(Altare et al., 2021; Ibrahim et al., 2021)* |
| **Society** | - Community engagement for trust building and supporting social capital *(Alameddine et al., 2019; Alonge et al., 2019; Atallah et al., 2018; Bang et al., 2020; Barker et al., 2020, 2020; Dean et al., 2020; Grimm et al., 2021; Kieny & Dovlo, 2015; Lancet, 2014; Ling et al., 2017; Miller et al., 2018)* - Community engagement for ownership of responses *(Barker et al., 2020; Kruk et al., 2017; Ling et al., 2017; Miller et al., 2018; Peters, 2017)*, paying attention to use local languages *(Bang et al., 2020; Mayhew et al., 2021)* - Localisation of aid to ensure trust from communities (Mayhew et al., 2021) | - Engagement with community-based organizations to strengthen surveillance *(Alonge et al., 2019; Barker et al., 2020)* | - Integration of a social justice lens in health systems research and strengthening approaches *(Atallah et al., 2018)* |
| **Systems** | - Improvement of health policies and legislations *(McKenzie et al., 2016; Miller et al., 2018; Nyenswah, 2017)* - Investment on institutional capacity strengthening and resources stewardship *(Hanefeld et al., 2018; T. Martineau et al., 2017)* - Adoption of a decentralised decision-making systems *(Alameddine et al., 2019; Grimm et al., 2021; Hanefeld et al., 2018; Jamal et al., 2020; F. P. Martineau, 2016; McKenzie et al., 2016)* - Pre-existing partnerships and/or coordination mechanisms between governments and communities *(Alonge et al., 2019; Barker et al., 2020; Christensen et al., 2021)* - Availability of emergency preparedness and response plans *(Alameddine et al., 2019; Ibrahim et al., 2021; Kruk et al., 2015; Ling et al., 2017; Moitinho de Almeida, 2022)* - Establishment of reliable early warning systems *(Christensen et al., 2021; Kieny & Dovlo, 2015, 2015; Nyenswah, 2017; Sochas et al., 2017)* - Investment on digital health information management systems *(Alameddine et al., 2019)* | - Strengthening of inter-sectoral coordination during an emergency *(Hamadeh et al., 2021; Ibrahim et al., 2021; Jamal et al., 2020; Kruk et al., 2017; Ling et al., 2017; Moitinho de Almeida, 2022)* - Engagement in long-term partnerships *(Kieny & Dovlo, 2015; Moitinho de Almeida, 2022; Tjoflåt & Hansen, 2019)* - Engagement with the private sector in response coordination efforts *(Hanefeld et al., 2018; Kruk et al., 2017; Mangouri & Zangana, 2017)* - Flexibility in management of health operations *(Ager et al., 2015; Jamal et al., 2020)* - Improvement of variety and quality of data collection *(Mayhew et al., 2021)*, also through digital innovation *(Sochas et al., 2017)* - Improved information flow across levels of the health system and between communities and health systems *(Alonge et al., 2019)* - Regular revision and update of emergency preparedness and response plans *(Jamal et al., 2020)* | - Investment in operational research to promote evidence-based changes in systems *(Atallah et al., 2018; F. P. Martineau, 2016; McKenzie et al., 2016)* |
| **Stocks, supplies, and other inputs** | - Investment on lean supply chain *(Alameddine et al., 2019)* - Investment on laboratory systems *(Nyenswah, 2017)* - Pre-existing free drug supply *(Ager et al., 2015; Christensen et al., 2021)* | - Pooled humanitarian funds for increased coordination and improved accountability *(Hanefeld et al., 2018; Kruk et al., 2017; Mangouri & Zangana, 2017; Odhiambo et al., 2020)* - Increased external funding *(Kieny & Dovlo, 2015; Ling et al., 2017)* - Expansion of procurement network *(Alameddine et al., 2019)* and support of supply chains *(Bang et al., 2020; Ibrahim et al., 2021; Tjoflåt & Hansen, 2019)* - Increase distribution of supplies at facility level in anticipation of disruptions in supply chain *(Altare et al., 2021)* - Removal of financial barrier to access health care, e.g. through free care provision or subsidized packages of care *(Ager et al., 2015; Hamadeh et al., 2021)* | - Promote research and development pipelines of drugs, medical supplies, and vaccines through an equity lens *(Lancet, 2014; Peters, 2017)* |
| **Space and built environment** | - Investment on health infrastructure and its resilience (Atallah et al., 2018; Cristiano et al., 2021; Odhiambo et al., 2020) | - Establishment of additional redundancies in the infrastructural system to increase capacity *(Jamal et al., 2020; Moitinho de Almeida, 2022)* | - Resiliency-based ex-novo construction *(Cristiano et al., 2021)* or reconstruction *(Ibrahim et al., 2021)* of non-functional health facilities |
| **Staff / Human resources** | - High levels of commitment and motivation, rooted in both professional ethics and sense of belonging to the community, among human resources for health, including community health workers *(Ager et al., 2015; Alameddine et al., 2019; Bou-Karroum et al., 2020, 2020; Christensen et al., 2021; Drevin et al., 2019; Grimm et al., 2021; Hanefeld et al., 2018; Ibrahim et al., 2021; Jamal et al., 2020; Kieny & Dovlo, 2015; Mangouri & Zangana, 2017; Moitinho de Almeida, 2022; Nyenswah, 2017; Sripad et al., 2021)* - Task-shifting as an appropriate action to support continuity of service provision in the wake of staff depletion often observed in FCAS *(Alameddine et al., 2019; Altare et al., 2021; Atallah et al., 2018; Bou-Karroum et al., 2020; Ibrahim et al., 2021; Jamal et al., 2020; Witter et al., 2017)* - Psycho-social support interventions targeted at the health care workforce *(Altare et al., 2021; Bou-Karroum et al., 2020; Dean et al., 2020; Ibrahim et al., 2021; Jamal et al., 2020; Sripad et al., 2021)* | - Support to health care workforce development *(Altare et al., 2021; Bang et al., 2020; Bou-Karroum et al., 2020; Cristiano et al., 2021; Ibrahim et al., 2021; Kieny & Dovlo, 2015; Ling et al., 2017; Moitinho de Almeida, 2022; Nyenswah, 2017)*, including community health workers *(Sripad et al., 2021)* - Recruitment of female health workers to support MNCH care continuity *(McKenzie et al., 2016)* - Individual adaptations and coping strategies at group level for health staff *(Martineau et al., 2017; Witter et al., 2017)*, including peer-support *(Witter et al., 2017)* - Adoption of task-shifting where not yet existing *(Alameddine et al., 2019; Altare et al., 2021; Atallah et al., 2018; Bou-Karroum et al., 2020; Ibrahim et al., 2021; Jamal et al., 2020; Witter et al., 2017)* - Deployment of health care workers to affected areas where surge in demand is observed *(Ibrahim et al., 2021)* | - Address violence against health care workers both in data collection and in interventions *(Altare et al., 2021; Bou-Karroum et al., 2020)* |
| **Services** | - Investment on people-centred, quality health service delivery *(Christensen et al., 2021; Kieny & Dovlo, 2015; McKenzie et al., 2016; Nyenswah, 2017)* - Integrated systems of care rather than vertical approaches *(Hanefeld et al., 2018)* - Reprioritization of service delivery – e.g. suspension of elective surgery *(Moitinho de Almeida, 2022)* - Integrate pharmacies and private clinic in network of service provision *(Ibrahim et al., 2021)* | - Improvement in quality of clinical protocols and procedures (including IPC measures) *(Bang et al., 2020; Ling et al., 2017)* - Expansion of offer of services beyond vertical programs *(Mayhew et al., 2021)* - Change in clinical practice to adapt to the existing gaps *(Barker et al., 2020; Drevin et al., 2019; Tjoflåt & Hansen, 2019)* - Mobile clinics for service delivery *(Altare et al., 2021; Grimm et al., 2021; Jamal et al., 2020)* | - Change in program design to respond to emerging needs *(Alameddine et al., 2019; Altare et al., 2021; Jamal et al., 2020)* |

1. Munyuzangabo M, Khalifa DS, Gaffey MF, Kamali M, Siddiqui FJ, Meteke S, et al. Delivery of sexual and reproductive health interventions in conflict settings: a systematic review. BMJ Glob Health. 2020 Jul 21;5(Suppl 1):e002206. [↑](#footnote-ref-2)
2. Munyuzangabo M, Gaffey MF, Khalifa DS, Als D, Ataullahjan A, Kamali M, et al. Delivering maternal and neonatal health interventions in conflict settings: a systematic review. BMJ Glob Health. 2021 Feb 19;5(Suppl 1):e003750. [↑](#footnote-ref-3)
3. Meteke S, Stefopulos M, Als D, Gaffey MF, Kamali M, Siddiqui FJ, et al. Delivering infectious disease interventions to women and children in conflict settings: a systematic review. BMJ Glob Health. 2020 Apr 26;5(Suppl 1):e001967. [↑](#footnote-ref-4)
4. Shah S, Munyuzangabo M, Gaffey MF, Kamali M, Jain RP, Als D, et al. Delivering non-communicable disease interventions to women and children in conflict settings: a systematic review. BMJ Glob Health. 2020 Apr 27;5(Suppl 1):e002047. [↑](#footnote-ref-5)
5. The policy report from Bou-Karroum et al. is the only item included in the systematic review from the grey literature search. [↑](#footnote-ref-6)
